# A Liquid Density Standard Over Wide Ranges of Temperature and Pressure Based on Toluene

**DOI:** 10.6028/jres.113.005

**Published:** 2008-02-01

**Authors:** Mark O. McLinden, Jolene D. Splett

**Affiliations:** Physical and Chemical Properties Division National Institute of Standards and Technology, Boulder, CO 80305-3328; Statistical Engineering Division National Institute of Standards and Technology, Boulder, CO 80305-3328

**Keywords:** calibration, density, standard reference material, toluene, uncertainty

## Abstract

The density of liquid toluene has been measured over the temperature range −60 °C to 200 °C with pressures up to 35 MPa. A two-sinker hydrostatic-balance densimeter utilizing a magnetic suspension coupling provided an absolute determination of the density with low uncertainties. These data are the basis of NIST Standard Reference Material® 211d for liquid density over the temperature range −50 °C to 150 °C and pressure range 0.1 MPa to 30 MPa. A thorough uncertainty analysis is presented; this includes effects resulting from the experimental density determination, possible degradation of the sample due to time and exposure to high temperatures, dissolved air, uncertainties in the empirical density model, and the sample-to-sample variations in the SRM vials. Also considered is the effect of uncertainty in the temperature and pressure measurements. This SRM is intended for the calibration of industrial densimeters.

## 1. Introduction

The property of fluid density is a vital parameter in a multitude of industrial processes. These include the control of chemical processes and the metering of fuels and other commodity chemicals. Often, the process stream is sampled through an industrial densimeter for continuous, real-time determination of the density. Such densimeters are not absolute instruments—they must be regularly calibrated at the conditions of use with fluids of known density.

The work presented here utilizes an absolute fluid densimeter to establish the density of toluene as a function of temperature and pressure for use as a calibration standard. The measured density data are presented, but of more relevance for calibration purposes, the density data are also represented in terms of an empirical function relating temperature, pressure, and density.

Toluene has a number of advantages as a density standard: it is a stable chemical of relatively low toxicity; its density of 865 kg/m^3^ at ambient conditions is well matched to many applications; its freezing point of −95 °C and boiling point of 111 °C cover the range of many industrial processes. Toluene has a low surface tension compared to that of water, and it is relatively inexpensive. The National Institute of Standards and Technology (NIST) has sold a density Standard Reference Material (SRM^®^) based on toluene for many years, but the previous SRM was certified only at ambient conditions: 15 °C to 25 °C and normal atmospheric pressure.

This SRM certifies the density of a particular batch of toluene. This approach is preferred for high-accuracy calibrations over the alternative approach of measuring the density of “very pure” toluene for at least two reasons. First, a batch certification is directly traceable to NIST, and this is often a requirement for high-level calibration laboratories. Second, toluene in very high purities is difficult to obtain. High-quality commercial toluene (e.g., reagent grade or HPLC grade) is usually intended for use as a chemical precursor or as a solvent in various chemical analyses; it is certified to be free of contaminants (such as sulfur compounds) that would affect the analysis, but other impurities, such as closely related organic compounds, are often present. The use of “pure” toluene would greatly complicate the traceability of density and shift the problem to one of determining purity and/or the effects of impurities on the density.

This work describes the development of an extended-range SRM (designated as SRM 211d) for fluid density over wide ranges of temperature and pressure. First, the experimental principle, apparatus, and procedures are described. A description of the calibration procedures establishes traceability to SI quantities. The most significant uncertainties in the experimental determination of the fluid density are shown to arise from uncertainties in the sinker volumes, and the calibration of the sinker volumes is described in detail. Sections 4 and 5 present the results and a thorough analysis of the uncertainties.

## 2. Experimental Determination of Density

### 2.1 Experimental Principle

The two-sinker densimeter used in this work is described in detail by McLinden and Lösch-Will [1], and this general type of instrument is described by Wagner and Kleinrahm [2]. In the present densimeter two sinkers of nearly the same mass, surface area, and surface material, but very different volumes, are weighed separately with a high-precision balance while immersed in a fluid of unknown density. The fluid density *ρ* is given by

$$\rho =\frac{\left(m_{1}-m_{2}\right)-\left(W_{1}-W_{2}\right)}{V_{1}-V_{2}},$$ (1)

where *m* and *V* are the sinker mass and volume, *W* is the balance reading, and the subscripts refer to the two sinkers. The main advantage of the two-sinker method is that adsorption onto the surface of the sinkers, systematic errors in the weighing, and other effects that reduce the accuracy of most buoyancy techniques cancel.

A magnetic suspension coupling transmits the gravity and buoyancy forces on the sinkers to the balance, thus isolating the fluid sample (which may be at high pressure and/or extremes of temperature) from the balance. The central elements of the coupling are two magnets, one on each side of a nonmagnetic pressure-separating wall. The top magnet, which is an electromagnet with a ferrite core, is hung from the balance. The bottom (permanent) magnet is held in stable suspension with respect to the top magnet by means of a feedback control circuit making fine adjustments in the electromagnet current. The permanent magnet is linked with a lifting device to pick up a sinker for weighing. A mass comparator with a resolution of 1 μg and a capacity of 111 g is used for the weighings. Each sinker has a mass of 60 g; they are fabricated of tantalum and titanium and are both gold plated.

**Fig. 1:**
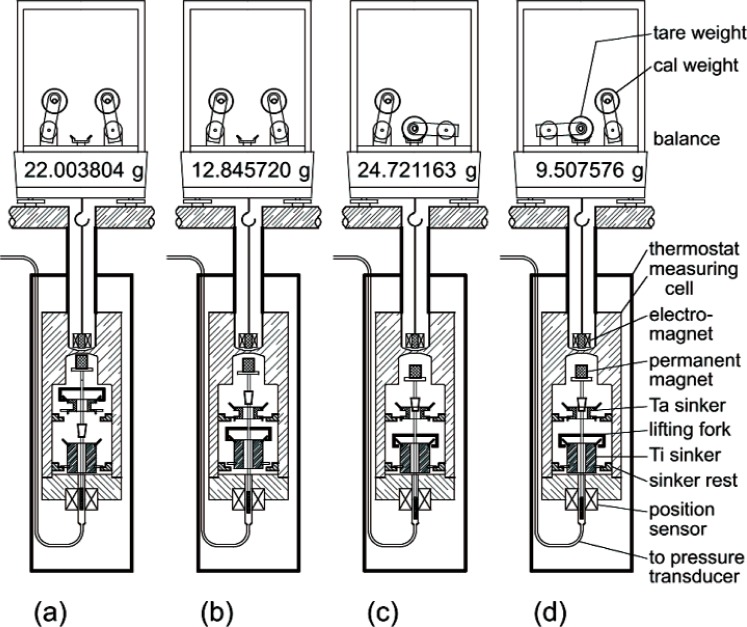
Schematic of the two-sinker densimeter showing the four weighings; (a) weighing of the tantalum sinker, (b) weighing of the titanium sinker, (c) weighing of the balance calibration weight, and (d) weighing of the balance tare weight; in (c) and (d) both sinkers are on their rests. Balance displays are typical for a fluid density of 941 kg/m^3^. Figure is not to scale.

Equation (1) must be corrected for magnetic effects; this is described by McLinden et al. [3]. In addition to the sinkers, two calibration masses are also weighed. Figure 1 shows a schematic of the density measuring cell and the four weighings. The weighings yield a set of four equations that are solved to yield, first, a balance calibration factor *α* and a parameter *β* related to the balance tare (i.e., those elements of the system that are always weighed):

$$\alpha =\frac{W_{\mathrm{cal}}-W_{\mathrm{tare}}}{\left(m_{\mathrm{cal}}-m_{\mathrm{tare}}\right)-\rho_{\mathrm{air}}\left(V_{\mathrm{cal}}-V_{\mathrm{tare}}\right)}\mathrm{and}$$ (2)

$$\beta =\frac{W_{\mathrm{cal}}}{\alpha }-\left(m_{\mathrm{cal}}-\rho_{\mathrm{air}}V_{\mathrm{cal}}\right).$$ (3)

where the subscripts cal and tare refer to the calibration weights; *ρ*_air_ is the density of the air (or purge gas) surrounding the balance and is calculated from the ambient temperature, pressure, and humidity measured in the balance chamber. In all the measurements reported here, the balance chamber was continuously purged with nitrogen. The “coupling factor” *ϕ*, which is the efficiency of the force transmission of the magnetic suspension coupling, is given by

$$\phi =\frac{\left(W_{1}/\alpha \right)-\beta }{m_{1}-\rho_{\mathrm{fluid}}V_{1}}.$$ (4)

Finally, the fluid density *ρ*_fluid_ is given by

$$\rho_{\mathrm{fluid}}=\frac{\left[\left(m_{1}-m_{2}\right)-\frac{\left(W_{1}-W_{2}\right)m_{1}}{W_{1}-\alpha \beta }\right]}{\left[\left(V_{1}-V_{2}\right)-\frac{\left(W_{1}-W_{2}\right)V_{1}}{W_{1}-\alpha \beta }\right]}-\rho_{0},$$ (5)

where *ρ*_0_ is the indicated density when measuring in vacuum. In other words *ρ*_0_ is an “apparatus zero,” which compensates for any changes in alignment or sinker masses. (The sinker masses were observed to change on the order of a few μg due to surface contamination and physical wear where they were picked up. Any shift in the alignment of the magnetic suspension coupling will result in a slight change in the apparent sinker masses.) The key point of the analysis by McLinden et al. [3] is that the density given by Eq. (5) compensates for the magnetic effects of both the apparatus and fluid being measured. With this apparatus, the coupling factor is nearly unity; for the present results it varied from 1.000 020 for vacuum to 0.999 975 for toluene at the highest density measured.

### 2.2 Apparatus Description

**Fig. 2:**
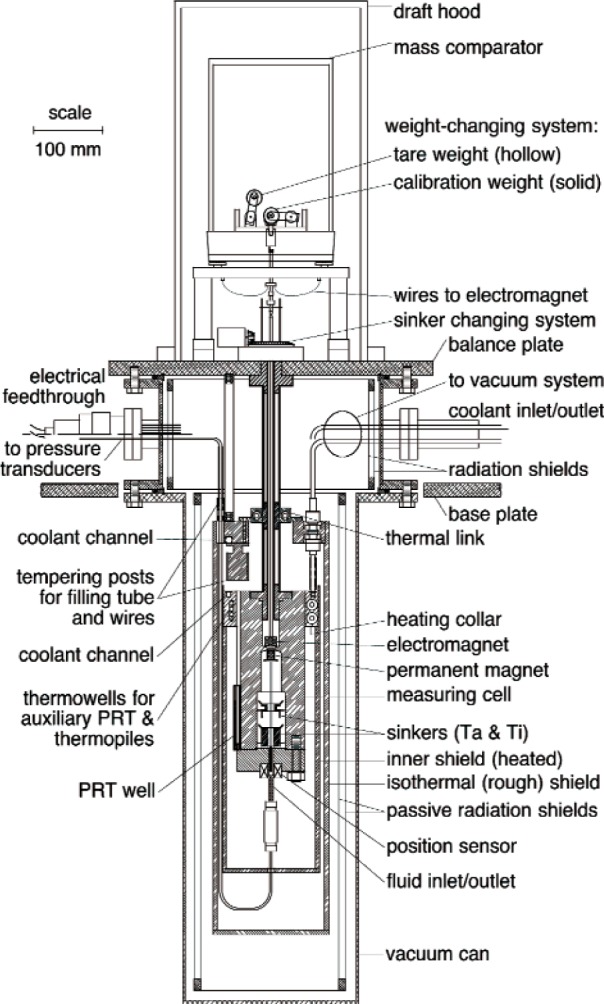
Detailed schematic of the density system and thermostat.

In addition to the sinkers, suspension coupling, and balance that make up the density measuring system, the apparatus includes a thermostat, pressure instrumentation, and a sample handling system. A schematic diagram of the densimeter is shown as Fig. 2.

The temperature is measured with a standard-reference-quality platinum resistance thermometer (SPRT) and resistance bridge referenced to a thermostatted standard resistor. The signal from the SPRT is used directly in a digital control circuit to maintain the cell temperature constant within ± 0.001 K. The pressures are measured with state-of-the-art transducers combined with careful calibration. The transducers (as well as the pressure manifold) are thermostatted to minimize the effects of variations in laboratory temperature.

The thermostat serves to isolate the measuring cell from ambient. It is a vacuum-insulated, cryostat-type design. The measuring cell is surrounded by an isothermal shield, which thermally isolates it from variations in ambient temperature; this shield was maintained at a constant (± 0.01 K) temperature 1 K below the cell temperature. Electric heaters compensate for the small heat flow from the cell to the shield and allow millikelvin-level control of the cell temperature. Operation at sub-ambient temperatures is effected by circulating ethanol from a chiller through channels in the shield.

### 2.3 Experimental Material

The material used is identical to the previous SRM, which is described as “a high purity liquid toluene … obtained from a commercial source.” [4] The SRM is provided in 5 mL flame-sealed glass ampoules. At the same time the 5 mL ampoules were prepared, several large 1.5 L flame-sealed ampoules were also prepared containing the same toluene. We worked with material from one of the 1.5 L ampoules, except for some of the chemical analysis, which used the 5 mL ampoules. We transferred the toluene from the 1.5 L ampoule to a 2.5 L stainless-steel sample cylinder for convenience in sample handling.

The sample was degassed by freezing the stainless-steel cylinder in liquid nitrogen, evacuating the vapor space, and thawing. The freeze/pump/thaw process was repeated a total of three times. The residual pressure over the frozen sample on the final cycle was 0.0002 Pa. The SRM as supplied by NIST contains some amount of dissolved air. The sample was degassed to obtain a well characterized state for the measurements. Also, we were concerned that dissolved air could react with the toluene at the elevated temperatures measured in this work. We felt that the uncertainties introduced by “purifying” the SRM material in this way would be offset by a reduction in possible effects resulting from reaction of the toluene with air. This point is discussed further in Sec. 4.3.

A chemical analysis by gas chromatography-mass spectrometry revealed the presence of trace levels of dimethyl benzenes and ethyl benzene; these are heavier impurities that would be expected to be present in toluene. A quantitative analysis by gas chromatography with a flame ionization detector yielded an overall purity of 99.92 % toluene with a standard uncertainty of 0.01 %. The sample was collected following the density measurements and reanalyzed; no significant differences were detected.

To quantify the effect of dissolved air on the density, additional measurements were made on a sample that was saturated with air at a temperature of 20 °C and pressure of 0.10 MPa. A quantity of the degassed toluene was transferred to an evacuated 500 mL stainless steel sample cylinder. Dry air was admitted to the cylinder to a pressure of 0.10 MPa; additional air was admitted periodically over the course of 24 hours to maintain the pressure at 0.10 MPa. The cylinder was periodically mixed to promote equilibrium. The air used was commercial “breathing air” with a moisture specification of 3 ppm. (Breathing air is air of normal atmospheric composition that has been dried.)

### 2.4 Experimental Procedures

Each density determination involved weighings in the order: tantalum (Ta) sinker, titanium (Ti) sinker, balance calibration weight, balance tare weight, balance tare weight (again), balance calibration weight, Ti sinker, and Ta sinker, for a total of eight weighings—two for each object. For each weighing, the balance was read five times over the course of ten seconds. For each object, the ten balance readings were averaged for use in Eqs. (2 to 5). Between each of the object weighings, and also before the first weighing and following the final weighing, the temperature and pressure were recorded, for a total of nine readings of *t* and *p*; these were also averaged. A complete density determination required 12 min. The weighing design was symmetrical with respect to time, and this tended to cancel the effects of any drift in the temperature or pressure.

The sample was loaded at a low temperature and pressure. Higher pressures were generated by heating the liquid-filled cell; this avoided the need for any type of compressor, which could have been a source of contamination, such as residual material from a previous test fluid. Starting at the lowest temperature and pressure for a given filling, measurements were made at increasing temperatures (and nearly constant density) until the maximum desired pressure was reached. The sample was then vented to a lower pressure along an isotherm.

The densimeter control program monitored the system temperatures and pressures once every 60 seconds. A running average and standard deviation of the temperatures and pressures were computed for the preceding eight readings. When these were within preset tolerances of the set-point conditions, a weighing sequence was triggered. Once the specified number of replicate density determinations were made at a given (*t,p*) state point, the control program then moved to the next temperature or automatically vented the sample to the next pressure on an isotherm.

Between each filling, and also before the first filling and following the last filling, the system was evacuated and the density recorded multiple times. The indicated density was used to determine the apparatus zero *ρ*_0_. The value of *ρ*_0_ used in Eq. (5) is the time-weighted average of *ρ*_0_ values measured before and after a given density determination.

## 3. Calibrations

### 3.1 Temperature and Pressure

The main platinum resistance thermometer (SPRT) used to measure the temperature of the fluid was calibrated on ITS–90 from 83 K to 505 K by use of fixed point cells (argon triple point, mercury triple point, water triple point, indium freezing point and tin freezing point). This was done as a system calibration, meaning that the SPRT was removed from its thermo-well in the measuring cell and inserted into the fixed point cell using the same lead wires, standard resistor, and resistance bridge that were used in the density measurements. The manufacturers of the fixed points have certified traceability to NIST and provide a temperature uncertainty of 1 mK or less. The fixed points and our calibration procedures were verified by checking each of the fixed point systems against a NIST-calibrated SPRT.

A full calibration of the main SPRT was carried out two months prior to the beginning of the toluene measurements. The resistance at the triple point of water was checked 16 months later; the resistance had changed by the equivalent of 0.5 mK. The standard (*k* = 1) uncertainty in the temperature, including uncertainty in the fixed point cells, drift in the SPRT, and temperature gradients between the SPRT and the actual fluid sample, is estimated to be 2 mK.

The pressure transducer was calibrated with a hybrid gas-oil piston gage system at pressures up to 40 MPa. Again, this calibration was done *in-situ* by connecting the piston gage to the sample port of the filling manifold. Based on the uncertainty for the piston gage, the repeatability observed for these transducers, and the uncertainties associated with the hydrostatic head correction, we estimate the standard uncertainty in pressure to be [(0.000026 · *p*)^2^ + (2.0 kPa)^2^]^0.5^, where the first term arises from uncertainties in the calibration, and the second term is a conservative estimate of the uncertainties arising from the head correction and the drift in the pressure transducer between calibrations.

### 3.2 Balance Calibration

An automated calibration of the mass comparator (i.e., the *α* in Eq. (2)) is an integral part of each density determination; it was achieved by a mechanism that lowers tare and calibration weights onto a modified balance pan. (The tare weight is required because the balance has a limited weighing range; without the tare weight, the balance would be “under-range.”) The weights were cylindrical in shape and fabricated of stainless steel (calibration weight) and hollow stainless steel (tare weight) with a mass difference of 15.2 g. The masses of these weights were determined by an SXXS-type comparison to standard masses [5]. The correction for air buoyancy on the standard mass was calculated by use of the BIPM air density equation [6] with ambient conditions measured with an electronic barometer and a temperature and humidity transmitter with the sensor located next to the balance.

The two weights were nearly identical in volume and surface area. The volumes of the calibration weights were determined by a simple hydrostatic determination using water as the density reference. Each volume was determined to be 7.4788 cm^3^. This provided a balance calibration that is very nearly independent of the density of the air or nitrogen purge gas surrounding the balance.

### 3.3 Sinker Volumes at 20 °C

Uncertainty in the sinker volumes was the major component of the overall fluid density uncertainty (as discussed in Sec. 5), and considerable effort was expended in determining these volumes accurately. The sinker volumes were determined using the hydrostatic comparator technique described by Bowman et al. [7,8]. This method differs from the traditional hydrostatic technique in that the known density is that of a solid object rather than that of a reference fluid, such as water. The standard and unknown objects are suspended in a fluid, but the fluid serves only to transfer the density knowledge of the standard to that of the unknown. The density of the fluid itself need not be known—it needs only to be constant during the period necessary to complete the measurement.

#### 3.3.1 Hydrostatic Apparatus

**Fig. 3:**
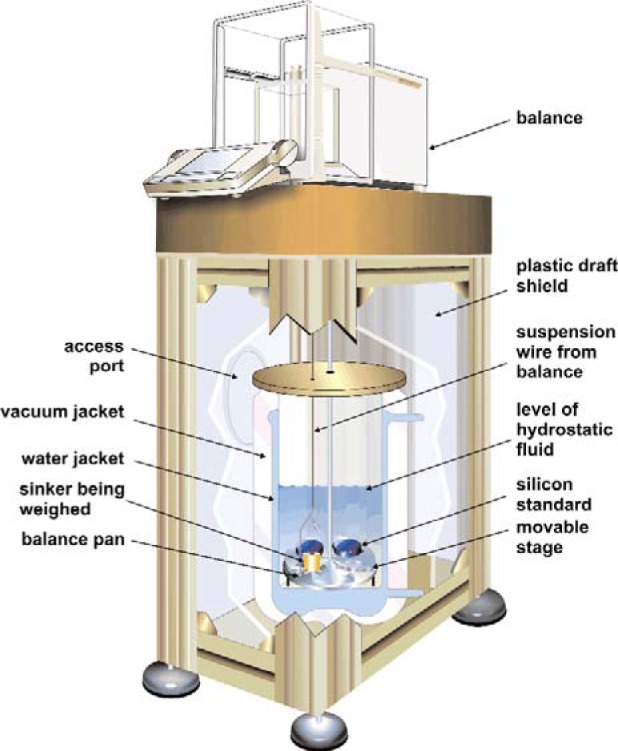
Cut-away view of the hydrostatic apparatus used to determine sinker volumes.

A separate apparatus has been developed at NIST to carry out the sinker volume determinations. It is modeled closely after the apparatus of Bowman et al. [7]. A thermostatted fluid bath contains a “stage” that allows the submerged objects to be placed one at a time onto a weighing “pan” that is suspended from the weighing hook of an analytical balance. The apparatus is shown in Fig. 3.

The fluid bath is a custom-built triple-walled beaker of borosilicate glass. The inner volume (approximately 170 mm inside diameter by 295 mm high) contains the hydrostatic fluid. It is surrounded by a water jacket connected to a circulating bath. The outermost jacket is evacuated for thermal insulation. A brass cover plate serves to minimize evaporation and temperature gradients. The bath is contained within a sturdy aluminum frame with plastic side panels to control drafts. The frame is topped by a 40 kg limestone block on which the balance sits.

The bath fluid is a high-density fluoroether (2-trifluoromethyl-3-ethoxydodecafluorohexane). This fluid has several advantages over water. Its high density of approximately 1631 kg/m^3^ increases the buoyancy force on the submerged objects and thus the sensitivity of the volume determination. Its lower surface tension (16 N/m compared to 73 N/m for water) decreases the forces on the suspension wire. This and the much higher gas solubility compared to that of water greatly reduce the problems associated with small air bubbles clinging to the objects.

The temperature of the fluid bath was measured with a standard-reference-quality SPRT in a thermowell located in close proximity to the weighing pan. The SPRT was calibrated on ITS–90 at the triple point of water (0.01 °C) and the melting point of gallium (29.7646 °C). The circulating bath was started at least 16 hours prior to the weighings to allow temperature equilibrium to be achieved. During the weighings, the standard deviation of the bath temperature was 1.7 mK, with a maximum deviation of 6 mK from the average value of 293.135 K.

The stage is a simple “turntable” that holds the objects to be weighed. It was manually lifted and rotated (using a central axle extending above the bath cover plate) to place the objects on the weighing pan. The weighing pan was suspended from the balance with a stainless steel wire 0.08 mm in diameter. The balance had a capacity of 205 g, resolution of 10 μg, and linearity of 30 μg. The balance was calibrated immediately before each determination with its built-in calibration weights and automatic calibration sequence. The balance was then checked with a 100 g standard mass (class E2; certified mass 99.999 94 ± 0.000 05 g). The balance reading was consistently low by an average of 0.14 mg, and an adjustment of 1.4 ppm was applied to all subsequent balance readings to compensate for this difference.

The standards are made of hyperpure, float-zone, single-crystal silicon. They are in the shape of right circular cylinders (49.8 mm diameter by 22.1 mm high) with a nominal mass of 100 g. Their densities were determined and certified by the NIST Mass Group [9] with an expanded (*k* = 2) uncertainty of 0.000 032 g/cm^3^, which is equal to 0.0014 % of their density of 2.329 095 g/cm^3^. This determination was carried out using techniques very similar to those described here. The density standards used by the NIST Mass Group were silicon crystals that are the U.S. national solid-density standards. In fact, they are the artifacts described by Bowman et al. [7], which are directly traceable to densities determined by dimensional measurements of near-perfect spheres by interferometry and mass measurements commencing with the U.S. national mass standards. Silicon is an ideal density standard because single-crystal material of very high purity is readily available at moderate cost. Its coefficient of thermal expansion and, thus, variation in density as a function of temperature, are known very well [10].

#### 3.3.2 Experimental Design

The hydrostatic apparatus accommodates four objects—two standards and two unknowns. This allows the simultaneous determination of the volumes of the tantalum and titanium sinkers and also provides the redundancy that permits a statistical analysis of the measurements. The experiment involves a series of A-B-A type weighings to yield ratios of the volumes of A and B. Bowman et al. [7] described a set of 15 weighings needed to determine six volume ratios. Here, the design was modified slightly to 16 weighings:

A-B-A-C-A-D-A-D-B-D-C-D-C-B-C-B,

where “A” is standard #1, “B” is the tantalum sinker, “C” is standard #2, and “D” is the titanium sinker. This design yields the ratios AB, AC, AD, DA, DB, DC, CD, CB and BC, or three more ratios than in the Bowman sequence, for only one additional weighing.

Each “weighing” in the experimental design consisted of the following steps:

1. Raise and rotate the stage to place the desired object into position above the weighing pan (this is defined as time 0:00).
2. Record the bath temperature and the balance reading for the empty weighing pan at time 8:00. (Rotation of the stage causes turbulence in the bath, and so several minutes were needed for this to subside.)
3. Lower the stage to place the object onto the weighing pan shortly after step 2 (approximately 8:30 to 9:00).
4. Record the bath temperature and the balance reading for the loaded weighing pan at time 16:00.

This sequence was repeated 15 more times (plus an additional weighing of the empty pan at the end) for a total elapsed time of 264 min. The thermometer and balance readings were recorded by computer within a few milliseconds of the specified times. This strict adherence to timing and the A-B-A design compensated for any linear drift in the balance zero and/or drift in the fluid density over the course of the experiment. The time between weighings was more than adequate to allow turbulence to subside (steady weighings were typically observed within three minutes of moving the stage). Also, the object was in the proximity of the SPRT for nearly 15 min before it was weighed, allowing time for temperature equilibration with the fluid in the vicinity of the SPRT. At the end of the complete weighing sequence the balance was tared (but not recalibrated) and again checked with the 100 g class E2 mass; the drift was less than 0.08 mg.

The masses of the sinkers and standards were determined at least twice on different days and the average value used in the analysis. A conventional mass determination in air was carried out using the balance. The correction for air buoyancy was calculated with the BIPM air density equation [6] with ambient conditions measured with an electronic barometer and a temperature and humidity transducer with the sensor located next to the balance.

Each balance weighing *W* is a summation of mass and buoyancy terms. For the empty pan

$$W_{\mathrm{pan}}=\left[m_{\mathrm{pan}}-\rho_{\mathrm{fluid}}V_{\mathrm{pan}}\right]/\left(1-\frac{\rho_{\mathrm{air}}}{\rho_{\mathrm{weights}}}\right),$$ (6)

where *m* is mass, *V* is volume and *ρ* is density. For the pan loaded with object “B”

$$W_{\text{pan}+B}=\frac{\left[m_{\mathrm{pan}}+m_{B}-\rho_{\mathrm{fluid}}\left(V_{\mathrm{pan}}+V_{B}\right)\right]}{\left(1-\frac{\rho_{\mathrm{air}}}{\rho_{\mathrm{weights}}}\right)}.$$ (7)

The (1 − *ρ*_air_/*ρ*_weights_) terms correct for air buoyancy— the balance was calibrated in air with stainless steel calibration masses with density *ρ*_weights_, but the submerged objects are not subject to air buoyancy. The air density in Eqs. (6) and (7) is that at the time of the balance calibration.

The average of the pan weighings immediately preceding and following each object weighing were subtracted from Eq. (7) to yield

$$W_{B}=\left[m_{B}-\rho_{\mathrm{fluid}}V_{B}\right]/\left(1-\frac{\rho_{\mathrm{air}}}{p_{\mathrm{weights}}}\right).$$ (8)

(Equations (6) to (8) are properly written in terms of force, not mass, since the balance used is a force transducer. But the acceleration of gravity cancels, and, by convention, the *m* × *g* force measured by the balance is recorded in terms of mass.)

Combining Eq. (8) with the average of two similar equations for the weighings of a second object immediately preceding and following the weighing of object B (i.e., the weighings of a A-B-A sequence) cancels the fluid density to yield the volume ratio:

$$\frac{V_{A}}{V_{B}}=\frac{m_{A}-W_{A}\left(1-\rho_{\mathrm{air}}/\rho_{\mathrm{weights}}\right)}{m_{B}-W_{B}\left(1-\rho_{\mathrm{air}}/\rho_{\mathrm{weight}}\right)}.$$ (9)

The measured volume ratios were determined at a temperature that differed slightly from the desired reference temperature of 20 °C. A small (maximum 0.26 ppm) correction was applied using literature values of the thermal expansion coefficient (Swenson [10] for silicon and Touloukian et al. [11] for tantalum and titanium) to adjust the volume ratios to the reference temperature.

#### 3.3.3 Results for Sinker Volumes

The volume ratios and resulting sinker volumes are given in Table 1. The experimental design provides a number of consistency checks. The repeat determinations of the volumes were very consistent, with a standard deviation of 0.000 003 cm^3^ for the tantalum sinker and 0.000 023 cm^3^ for the titanium sinker. Knowledge of the fluid density is not required, but the fluid density can be calculated from the results. (In fact, the apparatus serves as a highly sensitive single-sinker densimeter.) The fluid density was observed to have a nearly constant linear drift of 0.55 × 10^−6^ *ρ*/hr. This could be due to a drift in the balance calibration and/or absorption of air and water into the fluid, but in either case the effect was negligible over the 48 min required to complete an A-B-A weighing sequence.

The experimental design also yields the volume ratios of the two standards and of the two sinkers; these allow a further check of consistency. The measured volume ratio of the silicon standards (ratio AC) can be compared to the value calculated with the known values of mass and density. The directly measured ratio of the sinker volumes (ratio DB) can be compared to the value obtained from the volumes calculated from the other ratios. These are compared in Table 2 and are seen to be well within the expected uncertainties discussed below.

## 4. Results—Density of SRM 211d

### 4.1 Experimental Results

**Fig. 4:**
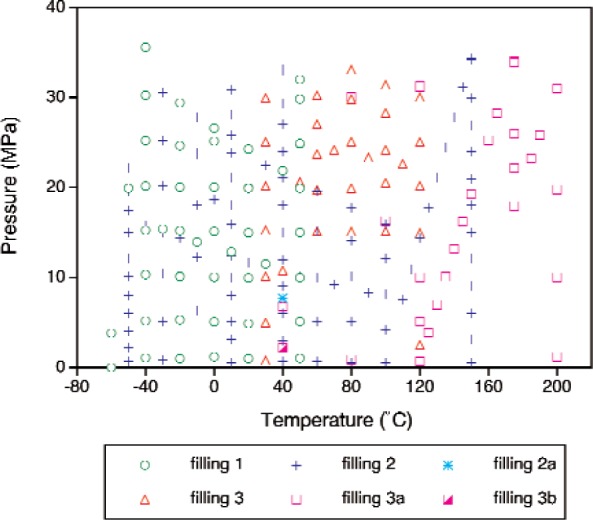
Temperature-pressure state points measured for the SRM toluene; the different symbols represent the different fillings.

The SRM toluene was measured at 195 separate temperature and pressure state points; at most state points, five repeat density determinations were carried out for a total of 975 *p-ρ* -*T* data points, as shown in Fig. 4. These measurements represent three separate fillings. The measurements proceeded from low temperature to high temperature for each filling, except that after high-temperature measurements had been completed for fillings 2 and 3, the sample was cooled and measured again at 40 °C. (This required adding a small quantity of fresh sample to the cell, and these are referred to as fillings 2a, 3a, and 3b.) This provided a check on consistency between the fillings and also on any possible degradation of the sample due to exposure to high temperatures. These measurements were carried out January thru March, 2006; the experimental points are given in Table A1 (see 7. Appendix A).

The effect of dissolved air was investigated with a separate set of measurements carried out in May 2007. An abbreviated set of measurements with the original (degassed) sample covered the temperature range − 40 °C to 150 °C, with pressures to 32 MPa. Selected replicate measurements were made at − 40 °C to 50 °C in a separate filling. The density was measured at 51 temperature and pressure state points with an average of four repeat density determinations per state point, for a total of 216 *p* - *ρ* - *T* data points. The air-saturated sample was then measured at similar temperatures and pressures at 40 temperature and pressure state points for a total of 180 *p* - *ρ* - *T* data points. Following the measurements at 150 °C, the sample was cooled to 50 °C and measured again. The data for the degassed sample are given in Table A2 and the air-saturated data are given in Table A3 (see 7. Appendix A).

### 4.2. Estimated Fluid Density

**Fig. 5:**
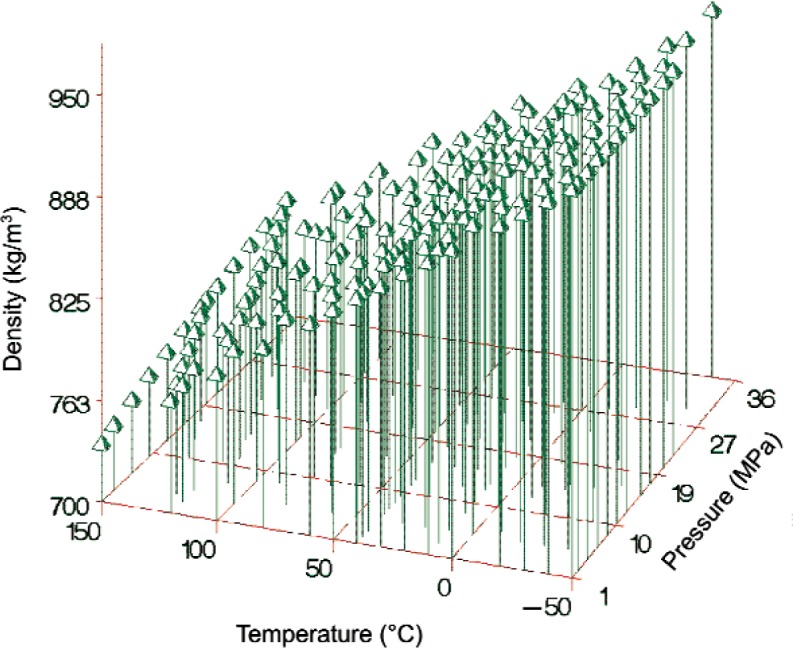
906 density measurements versus temperature and pressure used to develop the density model (Eq. 10).

The fluid density is represented using a 20-parameter empirical model

$$\rho =\sum_{k=1}^{8}a_{k}{\left(\frac{T}{100}\right)}^{-b_{k}}p^{c_{k}},$$ (10)

where *T* is temperature and *p* is pressure in MPa. (We use *T* to indicate temperatures in kelvins and *t* for temperatures in °C.) In fitting the model parameters, shown in Table 3, we excluded points with *t* < − 50 °C or *t* > 150 °C. The empirical model can be used to estimate the density for any temperature in the range of − 50 °C to 150 °C (223.15 K to 423.15 K) and any pressure in the range of 0.1 MPa to 30 MPa. The lower pressure limit represents a modest extrapolation of the experimental data; the upper pressure limit is conservative, since we used the data at *p* > 30 MPa in the fit. Table 4 gives values of *ρ*, calculated from Eq. (10) for even increments of temperature and pressure. Figure 5 displays the density measurements versus temperature and pressure that were used to fit the 20-parameter model.

### 4.3. Correction for Air-Saturated Samples

While the data used to fit the empirical model Eq. (10) were collected for degassed samples, the data measured at near-ambient conditions for the previous issue of this SRM were based on samples having some degree of air saturation. Thus, a correction *Δ* was added to the computed fluid density to align the near-ambient SRM and degassed data so that the estimated fluid density is

$$\rho_{\Delta }=\sum_{k=1}^{8}a_{k}{\left(\frac{T}{100}\right)}^{-b_{k}}p^{c_{k}}+\Delta =\rho +\Delta ,$$ (11)

where

$$\Delta =F_{\mathrm{air}}\cdot g,$$ (12)

and *Δ* is a function of *t* and *p.*

The value *F*_air_ represents the fraction of air saturation and *g* is the estimated density correction in kg/m^3^. If measurements are based on degassed samples, then *F*_air_ = 0, and the correction *Δ* and its associated uncertainty are zero.

The density correction for air-saturated samples was determined from the supplemental density measurements for both air-saturated and degassed samples (as listed in Tables A2 and A3). Because measurements for air-saturated and degassed samples could not be made at exactly the same temperatures and pressures, a rational function of the form

$$\rho_{\mathrm{saturated}}=\frac{a_{1}+a_{2}\ln\left(t\right)+a_{3}p+a_{4}{\left[\ln\left(t\right)\right]}^{2}+a_{5}p^{2}+a_{6}p\ln\left(t\right)}{1+a_{7}\ln\left(t\right)+a_{8}p+a_{9}{\left[\ln\left(t\right)\right]}^{2}+a_{10}p\ln\left(t\right)},$$ (13)

was fitted to the air-saturated density measurements, and a similar model was fitted separately to the degassed density measurements *ρ*_degassed_. Next, the two rational functions were used to predict the density of each point in the combined air-saturated and degassed data sets. The predicted density correction is

$$g={\hat{\rho }}_{\mathrm{saturated}}-{\hat{\rho }}_{\mathrm{degassed}},$$ (14)

where ${\hat{\rho }}_{\mathrm{saturated}}-{\hat{\rho }}_{\mathrm{degassed}}$ are the predicted densities based on each rational function.

**Fig. 6:**
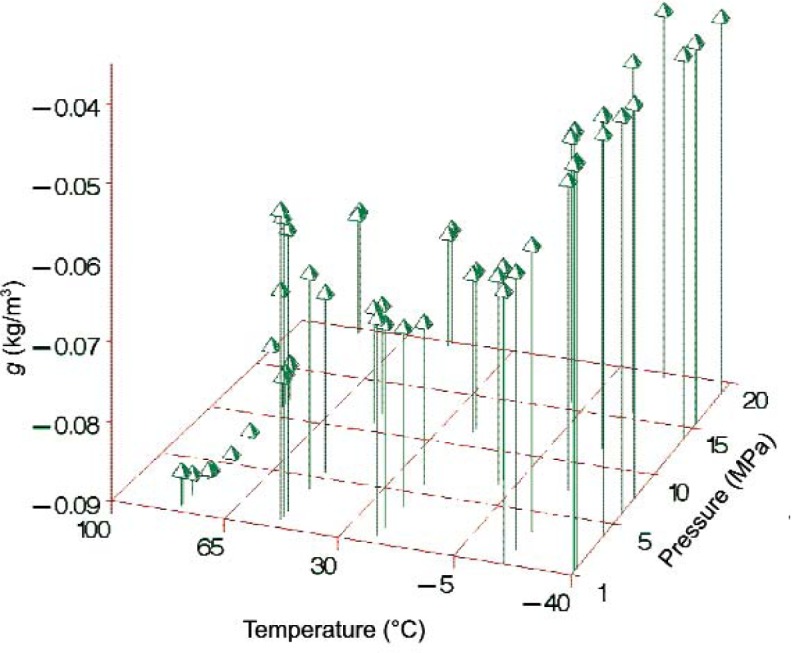
Density correction for air-saturated versus degassed toluene based on experimentally measured points.

**Fig. 7:**
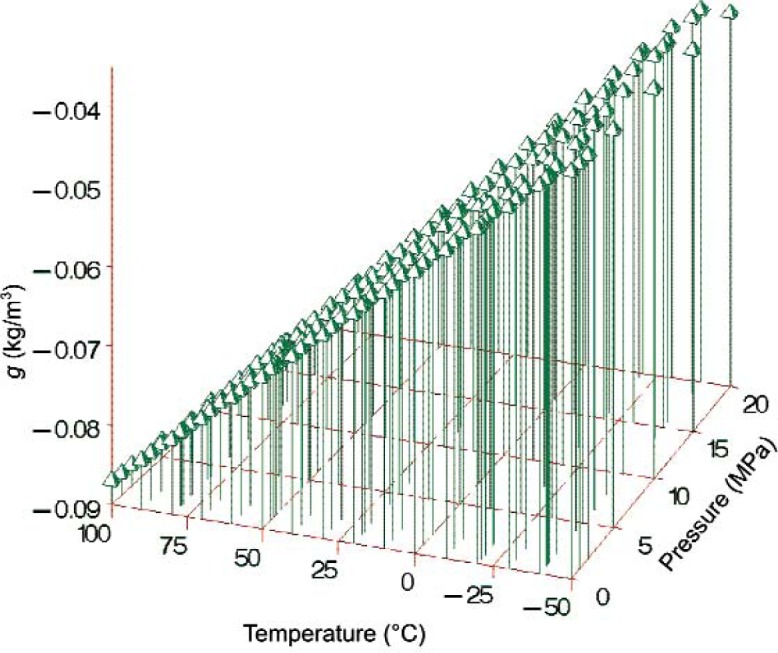
Estimated correction surface for air-saturated versus degassed toluene (Eq. 16).

We analyzed predicted corrections for pressures ranging from 0.1 MPa to 20 MPa and temperatures ranging from − 40 °C to 100 °C. The predicted correction surface for 228 different temperature and pressure combinations is shown in Fig. 6. We do not have a theoretical basis for selecting a functional form for the air-saturated density correction, and because this correction is only slightly larger than the uncertainties in the measured densities there is the danger of “over-fitting” with a strictly empirical function. Thus, we fitted a very simple temperature/pressure model

$$g={\hat{\rho }}_{\mathrm{saturated}}-{\hat{\rho }}_{\mathrm{degassed}}=b_{1}+b_{2}+t+b_{3}p+b_{4}tp$$ (15)

to the predicted corrections. Thus, the estimated correction for air-saturation is determined from

$$\begin{array}{l} g=-0.0549-3.1589\times 10^{-4}\cdot t+5.6019\times 10^{-5}\cdot p\\ \;-2.32\times 10^{-6}\cdot \left(t\cdot p\right), \end{array}$$ (16)

where *t* is in °C and *p* is in MPa. For example, the estimated density correction at 25 °C and 0.1 MPa is *g* = − 0.0628 kg/m^3^. This result is in excellent agreement with the value of − 0.062 kg/m^3^ ± 0.007 kg/m^3^ reported by Ashcroft and Isa [12] for degassed versus air-saturated toluene at similar conditions. Figure 7 displays the estimated correction surface for air-saturation based on Eq. (16) for selected temperatures and pressures. The correction is not reliable for pressures smaller than 0.1 MPa or larger than 20 MPa, and temperatures less than − 50 °C or greater than 100 °C.

We measured the air-saturated samples at temperatures up to 150 °C, but in fitting Eq. (16) we found that the trends in *g* with temperature and pressure became inconsistent at temperatures above 100 °C. We took this as evidence of decomposition and/or reaction of the toluene with oxygen at the higher temperatures. Thus, we fitted the correction only to the lower-temperature data. The lower temperature limit of − 50 °C for Eq. (16) represents a modest extrapolation of the experimental data.

The densities measured in May 2007 for the degassed toluene are, on average, 0.057 kg/m^3^ higher than the densities measured during January to March 2006. The 2006 measurements (which form the basis for the “official” SRM densities) were also made on the same (degassed) sample. This difference is larger than the standard uncertainty in the measured densities, although it is within the expanded uncertainty. The sample had been stored in the 2.5 L stainless steel sample cylinder in the 13 months between the two series of measurements. Storage in a metal container may result in more degradation of the sample, compared to storage in the glass SRM vials. There is also the possibility that the densimeter drifted by this amount. We carry out periodic measurements on high-purity argon to check for any such drifts, and we found no significant differences between argon measurements made in January 2005 and those made in June 2007.

## 5. Uncertainty Analysis

The overall uncertainty in the fluid density arises from several distinct sources. The first source is the empirical model used to represent the density and allow interpolation at a desired temperature and pressure. A second category relates to the material itself; these include uncertainties associated with the degree of air saturation of the toluene and possible degradation resulting from exposure to high temperatures. Since the SRM is provided in 5 mL ampoules (vials), the variation in density from vial to vial must also be considered. The third, and most complex, source arises from the experimental measurement of the density. Finally, when using the SRM for the calibration of a densimeter, the uncertainty in the *user’s* temperature and pressure measurement must be included.

According to accepted methods for determining uncertainty [13], the measurement equation is the starting point for estimating uncertainty. For practical purposes, our measurement equation is given by Eq. (11), however vial-to-vial effects (*V*), apparatus effects (*e*), material degradation effects (*x*), and errors in the user’s temperature and pressure measurements (*tp*) must also be included in the measurement equation even though their values are thought to be zero. The complete measurement equation is thus

$$\rho_{C}=\rho +\Delta +V+x+e+tp,$$ (17)

where *ρ* represents the density estimate from the empirical model and *Δ* is the correction for air saturation. Although the values of *V*, *x*, *e*, and *tp* are thought to be zero, they still have some uncertainty.

The combined standard uncertainty, assuming independent input quantities, for the estimated fluid density *ρ_C_* Eq. (17) is

$$u_{C}={\left[u^{2}\left(\rho \right)+u^{2}\left(\Delta \right)+u^{2}\left(V\right)+u^{2}\left(x\right)+u^{2}\left(e\right)+u^{2}\left(tp\right)\right]}^{0.5},$$ (18)

where *u*(*ρ*) is the uncertainty associated with the empirical model, *u*(*V*) is the uncertainty associated with vial-to-vial variations (based on measurements carried out at near-ambient conditions), and *u*(*x*) is the uncertainty associated with any possible degradation (i.e., change in chemical composition) of the sample resulting from exposing it to high temperatures. The uncertainty associated with the “air-saturated” correction is *u*(*Δ*). If samples are degassed before taking measurements, then no correction is needed and *u*(*Δ*) = 0. The quantity *u*(*e*) is the uncertainty associated with a single experimental density measurement, which we can think of as a method/apparatus uncertainty. The final uncertainty component *u*(*tp*) represents the uncertainty associated with the *user’s* temperature and pressure measurements.

Details regarding the estimation of each of these uncertainty components are provided below.

### 5.1 Uncertainty *u*(*ρ*) Due to Empirical Model

Our best estimate of the uncertainty associated with the 20-parameter empirical model to fit density versus temperature and pressure is the root-mean-squared error of the fit, or

$$u\left(\rho \right)={\left[\frac{\sum_{i=1}^{n}{\left(\rho_{i}-\rho_{\mathrm{fit}}\right)}^{2}}{n-p}\right]}^{0.5},$$ (19)

where *n* is the number of observations used in the fit, *p* is the number of parameters estimated, *ρ_i_* denotes the *i* th observation of density, and *ρ*_fit_ is the fitted value associated with the *i* th observation. The value of *u*(*ρ*) is 0.0086 kg/m^3^ for our fit, and there are 906 − 20 = 886 degrees of freedom associated with *u*(*ρ*) (*df_ρ_* = 886).

### 5.2 Uncertainty *u*(*Δ*) Due to Air-Saturation of Samples

Based on the air-saturation correction equation *Δ* = *F*_air_ · g, the standard uncertainty associated with *Δ* (Eq. 12) is

$$\begin{array}{l} u\left(\Delta \right)={\left[{\left(\frac{\partial \Delta }{\partial F_{\mathrm{air}}}\right)}^{2}u^{2}\left(F_{\mathrm{air}}\right)+{\left(\frac{\partial \Delta }{\partial g}\right)}^{2}u^{2}\left(g\right)\right]}^{0.5}\\ \;={\left[g^{2}u^{2}\left(F_{\mathrm{air}}\right)+F_{\mathrm{air}}^{2}u^{2}\left(g\right)\right]}^{0.5}, \end{array}$$ (20)

obtained by use of propagation of errors techniques and by assuming that *F*_air_ and *g* are independent. The value of *u*(*Δ*) depends on both input quantities as well as their associated uncertainties. If the user is taking measurements on degassed samples, then *Δ* = 0 and *u*(*Δ*) = 0. The standard uncertainty associated with the correction *g* is *u*(*g*) = 0.0075 kg/m^3^, based on the worst-case prediction error associated with the model fit, i.e. Eq. (16). The degrees of freedom associated with *u*(*g*) are *df_g_* = 224, based on 228 (*t,p*) data points and four model parameters.

The value of *F*_air_ for the SRM samples is estimated to be 0.59. We will assume the error in *F*_air_ is uniformly distributed within the interval 0.49 to 0.69 so that the standard uncertainty of *F*_air_ is

$$u\left(F_{\mathrm{air}}\right)=\frac{0.69-0.49}{2\sqrt{3}}=0.058.$$ (21)

Assuming that the “uncertainty of the uncertainty” is 25 %, eight degrees of freedom are appropriate for the uncertainty due to *F*_air_ (*dfF*_air_ = 8). (See equation G.3 of [13] for details regarding the degrees of freedom approximation.)

The degrees of freedom associated with *u*(*Δ*) are

$$df_{\Delta }=\frac{u^{4}\left(\Delta \right)}{\frac{{\left[g\cdot u\left(F_{\mathrm{air}}\right)\right]}^{4}}{df_{F_{\mathrm{air}}}}+\frac{{\left[F_{\mathrm{air}}\cdot u\left(g\right)\right]}^{4}}{df_{g}}},$$ (22)

based on the Welch-Satterthwaite approximation [13]. The values of *Δ* and *u*(*Δ*) for the SRM at (20 °C, 0.10 MPa, and *F*_air_ = 0.59) are *Δ* = − 0.0361 kg/m^3^ and *u*(*Δ*) = 0.0054 kg/m^3^ with *df*_Δ_ = 25.

### 5.3 Uncertainty *u*(*V*) Due to Vial-to-Vial Variability at Near-Ambient Conditions

The value of *u*(*V*) represents the combined vial-to-vial, day-to-day, and apparatus uncertainties at near-ambient conditions provided in the previous SRM report of analysis [4]. This analysis involved using a vibrating-tube densimeter to compare the density of samples from randomly selected 5 mL ampoules with the toluene used in the hydrostatic apparatus described by Bean and Houser [4]. The three sources of uncertainty included in *u*(*V*), and their degrees of freedom, are listed in Table 5 for convenience. The value of *u*(*V*) = 0.0114 kg/m^3^ was determined by adding the three sources in quadrature and taking the square root of the sum. The degrees of freedom, *df_V_* = 32, were calculated with the Welch-Satterthwaite approximation. We assume that *u*(*V*) is the same for all temperatures and pressures.

### 5.4 Uncertainty Due to Material Degradation and Time *u*(*x*)

**Fig. 8:**
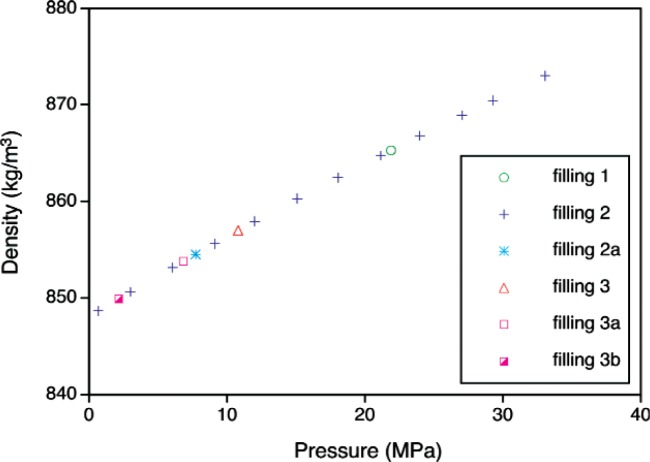
Replicate measurements at 40 °C for the various fillings of toluene.

Replicate measurements collected at 40 °C at the completion of a filling were used to determine the uncertainty due to material degradation and time effects. Any degradation in the toluene would be expected to be a function of both time and temperature (a short time at a high temperature would yield degradation similar to that resulting from a prolonged exposure to a moderate temperature). This term is also confounded with any possible drift in the experimental apparatus with time. While repeat measurements for a target temperature are easily obtained, target pressures are more difficult to achieve, and thus the pressures vary among the replicates. Figure 8 displays measurements taken at 40 °C over the course of this study.

**Fig. 9:**
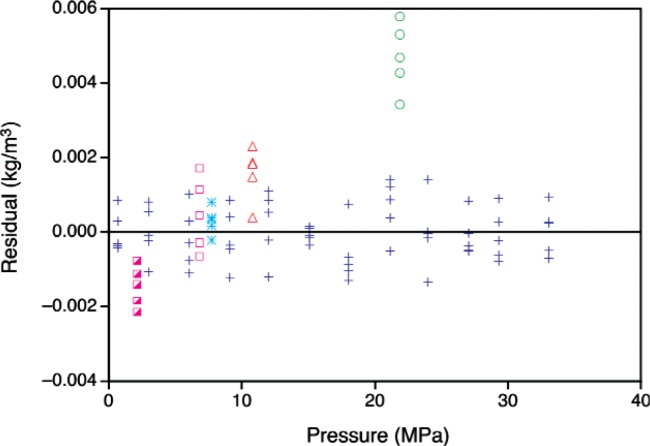
Residuals from the polynomial fit to filling #2 data at 40 °C; the plot symbols are the same as in Fig. 8.

A fourth-order polynomial was fitted to the “complete” 40 °C isotherm for the filling #2 data in Fig. 8; these measurements were made before the sample was exposed to higher temperatures. The residuals from the fit are shown in Fig. 9. The data from other fillings provide information about how the material may have changed over time and/or with exposure to high temperatures. The residuals indicate that the data from the other fillings are similar to the filling #2 data, with the exception of the filling #1 measurements at about 22 MPa. We will assume that the largest residual (conservatively estimated at 0.006 kg/m^3^) represents the worst-case error that might be observed. If the worst-case error is also assumed to represent the bounds of a uniform distribution, (− 0.006 kg/m^3^, 0.006 kg/m^3^) we can approximate the uncertainty due to material degradation and time effects as

$$u\left(x\right)=\frac{0.006\mathrm{kg}/m^{3}}{\sqrt{3}}=0.0035\;\mathrm{kg}/m^{3}.$$ (23)

This uncertainty is assumed to be valid for all temperatures included in this study. Assuming that the “uncertainty of the uncertainty” is 25 %, eight degrees of freedom are appropriate for the uncertainties due to material degradation and time errors (*df*_x_ = 8).

### 5.5 Uncertainty *u* (*e*) Due to Method/Apparatus

To estimate *u* (*e*) for given values of temperature (°C) and pressure (MPa), the polynomial equation

$$u\left(e\right)=\left\{\begin{array}{c} 0.02670+2.064\times 10^{-6}\cdot t+2.468\times 10^{-6}\cdot t^{2}\\ -1.88661\times 10^{-8}\cdot t^{3}\\ +4.56257\times 10^{-11}\cdot t^{4}+4.662\times 10^{-5}\cdot p\\ +3.41\times 10^{-6}\cdot p^{2} \end{array}\right\}$$ (24)

was fitted to values of the uncertainty associated with each measurement of fluid density across the temperature-pressure surface. Details regarding the computation of the uncertainty of fluid density measurements *u* (*ρ*_fluid_) are discussed in the sections that follow. The quantity *u* (*e*) denotes the uncertainty *predicted* by the polynomial based on the values of *u* (*ρ*_fluid_) computed for each data point. The error introduced into *u* (*e*) by using the polynomial equation is negligible compared to the magnitude of *u* (*e*). The degrees of freedom associated with *u* (*ρ*_fluid_) vary depending on pressure and temperature, and range from 9 to 18. Thus, a conservative estimate for the degrees of freedom associated with *u* (*e*) is 9 (*df_e_* = 9).

#### 5.5.1 Uncertainty *u*(*ρ*_fluid_) in Fluid Density Measurements

As discussed in Sec. 2, the experimental fluid density data were calculated with Eqs. (2 to 5). The uncertainty of a single fluid density measurement, *ρ*_fluid_, thus, is a function of the 14 input quantities:

$$\begin{array}{c} u\left(\rho_{\mathrm{fluid}}\right)=f(m_{\mathrm{cal}},m_{\mathrm{tare}},m_{1},m_{2},V_{\mathrm{cal}},V_{\mathrm{tare}},V_{1},V_{2},\\ \rho_{N_{2}},W_{\mathrm{cal}},W_{\mathrm{tare}},W_{1},W_{2},\rho_{0}). \end{array}$$ (25)

Many of the 14 input quantities are, in turn, dependent upon other measured quantities.

The values of *u* (*ρ*_fluid_) computed for each data point will be used to determine the parameters of a polynomial to predict uncertainty for a given temperature and pressure. The predicted uncertainty, *u*(*e*), represents the method/apparatus error. Based on propagationof errors techniques, assuming that all the input quantities are independent, the variance of *ρ*_fluid_ is

$$\begin{array}{l} u^{2}(\rho_{\text{fluid}})={\left(\frac{\partial \rho_{\mathrm{fluid}}}{\partial m_{\mathrm{cal}}}\right)}^{2}u^{2}\left(m_{\mathrm{cal}}\right)+{\left(\frac{\partial \rho_{\mathrm{fluid}}}{\partial m_{\mathrm{tare}}}\right)}^{2}u^{2}\left(m_{\mathrm{tare}}\right)\\ \;+{\left(\frac{\partial \rho_{\mathrm{fluid}}}{\partial m_{1}}\right)}^{2}u^{2}\left(m_{1}\right)+{\left(\frac{\partial \rho_{\mathrm{fluid}}}{\partial m_{2}}\right)}^{2}u^{2}\left(m_{2}\right)\\ \;+{\left(\frac{\partial \rho_{\mathrm{fluid}}}{\partial V_{\mathrm{cal}}}\right)}^{2}u^{2}\left(V_{\mathrm{cal}}\right)+{\left(\frac{\partial \rho_{\mathrm{fluid}}}{\partial V_{\mathrm{tare}}}\right)}^{2}u^{2}\left(V_{\mathrm{tare}}\right)\\ \;+{\left(\frac{\partial \rho_{\mathrm{fluid}}}{\partial V_{1}}\right)}^{2}u^{2}\left(V_{1}\right)+{\left(\frac{\partial \rho_{\mathrm{fluid}}}{\partial V_{2}}\right)}^{2}u^{2}\left(V_{2}\right)\\ \;+{\left(\frac{\partial \rho_{\mathrm{fluid}}}{\partial \rho_{N2}}\right)}^{2}u^{2}\left(\rho_{N2}\right)+{\left(\frac{\partial \rho_{\mathrm{fluid}}}{\partial W_{\mathrm{cal}}}\right)}^{2}u^{2}\left(W_{\mathrm{cal}}\right)\\ \;+{\left(\frac{\partial \rho_{\mathrm{fluid}}}{\partial W_{\mathrm{tare}}}\right)}^{2}u^{2}\left(W_{\mathrm{tare}}\right)+{\left(\frac{\partial \rho_{\mathrm{fluid}}}{\partial W_{1}}\right)}^{2}u^{2}\left(W_{1}\right)\\ \;+{\left(\frac{\partial \rho_{\mathrm{fluid}}}{\partial W_{2}}\right)}^{2}u^{2}\left(W_{2}\right)+{\left(\frac{\partial \rho_{\mathrm{fluid}}}{\partial \rho_{0}}\right)}^{2}u^{2}\left(\rho_{0}\right) \end{array}$$ (26)

and the combined standard uncertainty is the square root of the variance.

The Welch-Satterthwaite approximation [13] was used to estimate the degrees of freedom associated with *u* (*ρ*_fluid_):

$$df_{\mathrm{fluid}}=\frac{u^{4}\left(\rho_{\mathrm{fluid}}\right)}{D},$$ (27)

where

$$D=\sum_{i}\frac{{\left(\frac{\partial \rho_{\mathrm{fluid}}}{\partial \Psi_{i}}\right)}^{4}u^{4}\left(\Psi_{i}\right)}{df_{\Psi_{i}}},$$ (28)

and *Ψ_i_* represents each of the 14 input variables in Eq. (25). The derivatives in *u* (*ρ*_fluid_) and *df*_fluid_ are quite complicated, so we used a commercial symbolic algebra software package to generate the derivatives.

Next, we will provide details regarding the estimation of each individual component of uncertainty and its associated degrees of freedom.

#### 5.5.2 Uncertainties *u* (*m*cal), *u* (*m*tare), *u* (*m*_1_), and *u* (*m*_2_) in Masses

A single measurement of the mass of an unknown object, *m*_xi_, is determined by comparison to standard masses using a “SXXS” method (with S referring to a standard and X the unknown). By this method the mass of the unknown is given by

$$\begin{array}{c} m_{xi}=\{m_{s}\left(1-\frac{\rho_{\mathrm{air}}}{\rho_{s}}\right)+\left[\frac{\left(O_{2}-O_{1}\right)+\left(O_{3}-O_{4}\right)}{2\left(O_{3}-O_{2}\right)}\right]\\ m_{sw}\left(1-\frac{\rho_{\mathrm{air}}}{\rho_{\mathrm{sw}}}\right)\}/\left(1-\frac{\rho_{\mathrm{air}}}{\rho_{x}}\right), \end{array}$$ (29)

where the *O_i_* are the balance readings, the *m*_s_ and *m*_sw_ are standard masses, and *ρ*_s_ and *ρ*_sw_ are the densities of the standard masses. This method is described as “Standard Operating Procedure 4” by Harris and Torres [5]. We need to estimate the mass and the uncertainty of the mass for two sinkers, *m*_1_ and *m*_2_. We will assume the uncertainties associated with the calibration masses *m*_cal_ and *m*_tare_ are the same as those for *m*_1_ and *m*_2_.

Propagation of errors was used to determine the uncertainty associated with a single mass measurement. Assuming all input quantities are independent, the variance of the unknown mass is

$$\begin{array}{l} u^{2}\left(m_{xi}\right)={\left(\frac{\partial m_{xi}}{\partial m_{s}}\right)}^{2}u^{2}\left(m_{s}\right)+{\left(\frac{\partial m_{xi}}{\partial m_{\mathrm{sw}}}\right)}^{2}u^{2}\left(m_{\mathrm{sw}}\right)\\ +{\left(\frac{\partial m_{xi}}{\partial \rho_{\mathrm{air}}}\right)}^{2}u^{2}\left(\rho_{\mathrm{air}}\right)+{\left(\frac{\partial m_{xi}}{\partial \rho_{s}}\right)}^{2}u^{2}\left(\rho_{s}\right)\\ +{\left(\frac{\partial m_{xi}}{\partial \rho_{\mathrm{sw}}}\right)}^{2}u^{2}\left(\rho_{\mathrm{sw}}\right)+{\left(\frac{\partial m_{xi}}{\partial \rho_{x}}\right)}^{2}u^{2}\left(\rho_{x}\right)\\ +{\left(\frac{\partial m_{xi}}{\partial {Ο}_{1}}\right)}^{2}u^{2}\left({Ο}_{1}\right)+{\left(\frac{\partial m_{xi}}{\partial {Ο}_{2}}\right)}^{2}u^{2}\left({Ο}_{2}\right)\\ +{\left(\frac{\partial m_{xi}}{\partial {Ο}_{3}}\right)}^{2}u^{2}\left({Ο}_{3}\right)+{\left(\frac{\partial m_{xi}}{\partial {Ο}_{4}}\right)}^{2}u^{2}\left({Ο}_{4}\right) \end{array}$$ (30)

and the combined standard uncertainty of the unknown mass is *u* (*m*_x_*_i_*) = [*u*^2^(*m*_x_*_i_*)]^0.5^.

Next, we describe the evaluation of each individual uncertainty component in the mass determination. Table 6 displays information for the standard masses provided by their manufacturer’s calibration laboratory. Since the nominal value of *m*_s_ = 60 g is obtained by use of the 50 g and 10 g standards together, the uncertainty of *m*_s_ is

$$\begin{array}{l} u\left(m_{s}\right)={\left[{\left(0.000\;011\;45\;g\right)}^{2}+{\left(0.000\;008\;15\;g\right)}^{2}\right]}^{0.5}\\ \;=0.000\;014\;g. \end{array}$$ (31)

The nominal value of *m*_sw_ is 2 g, so *u* (*m*_sw_) = 0.000 0032 g. We believe the errors associated with the density of the standard masses are best described by a uniform distribution bounded by 0.05 g/cm^3^. Thus standard uncertainties of *ρ*_s_ and *ρ*_sw_ are

$$u\left(\rho_{s}\right)=u\left(\rho_{\mathrm{sw}}\right)=\frac{0.05\;{g/\mathrm{cm}}^{3}}{\sqrt{3}}=0.029\;{g/\mathrm{cm}}^{3}.$$ (32)

We know from the analysis of the sinker volume determination (Secs. 3.3 and 5.5.4) that the standard uncertainties associated with the densities of *m*_1_ and *m*_2_ used in our experiment are *u* (*ρ*_x_) = 0.000 11 g/cm^3^ for the density of sinker 1 (titanium) and *u* (*ρ*_x_) = 0.000 42 g/cm^3^ for the density of sinker 2 (tantalum). The repeatability standard deviation of the balance is 0.03 mg or 0.000 03 g, so the standard uncertainties of the observed balance readings are *u* (*O*_1_) = *u* (*O*_2_) = *u* (*O*_3_) = *u* (*O*_4_) = 0.000 03 g.

A single determination of the density of moist air, *ρ*_air_, was computed using the function of Davis [6], which is ultimately a function of temperature (*t*), pressure (*p*), and relative humidity (*h*). Using propagation of errors, and assuming independence of input quantities, the combined standard uncertainty of a single measurement of *ρ*_air_ is

$$\begin{array}{l} u\left(\rho_{\mathrm{air}}\right)=[{\left(\frac{\partial \rho_{\mathrm{air}}}{\partial t}\right)}^{2}u^{2}\left(t\right)+{\left(\frac{\partial \rho_{\mathrm{air}}}{\partial p}\right)}^{2}u^{2}\left(p\right)\\ \;+{{\left(\frac{\partial \rho_{\mathrm{air}}}{\partial h}\right)}^{2}u^{2}\left(h\right)]}^{0.5}. \end{array}$$ (33)

For a single determination of air density, the standard uncertainties for temperature, pressure, and humidity are *u* (*t*) = 0.2 K, *u* (*p*) = 0.0001 ⋅ *p* kPa, and *u* (*h*) = 0.02.

The value *u* (*m*_x_*_i_*) is the uncertainty associated with a single mass determination. The nominal mass values used in the density calculations are averages based on six repeat measurements (three measurements on each of two days), so we need to determine the uncertainty of the average mass.

Typically we would use the six repeat measurements to determine the uncertainty of the average mass value; however, a more extensive repeatability study was performed over four days with three repeated measurements per day. Thus, we will estimate the uncertainty of the average mass using the larger, more comprehensive data set and assume the uncertainty will be the same for the six measurements actually used. There appears to be no significant between-day effect for either sinker based on an analysis of variance, so we were able to combine all data and ignore the fact that the measurements were taken on different days.

We need to estimate two sources of variation—within measurements and between measurements—from the larger repeatability study in order to compute the uncertainty of the average mass. The within-measurement variance was computed as the average variance of the 12 repeated measurements,

$$u^{2}\left(m_{w}\right)=\frac{1}{12}\sum_{i=1}^{12}u^{2}\left(m_{x\;i}\right).$$ (34)

The values of *u* ^2^(*m*_x_*_i_*) were computed as described earlier in this section. The between-measurement variance is computed as the variance of the 12 mass measurements

$$u^{2}\left(m_{b}\right)=\frac{1}{12-1}\sum_{i=1}^{12}{\left(m_{x\;i}-{\bar{m}}_{x}\right)}^{2}.$$ (35)

Assuming the within-measurement and between-measurement variation based on the larger repeatability study are the same for the six measurements actually used in the experiment, the uncertainty of the average mass based on six observations is

$$u\left(m_{x}\right)={\left[\frac{u^{2}\left(m_{w}\right)}{6}+u^{2}\left(m_{b}\right)\right]}^{0.5}.$$ (36)

The estimated uncertainties for sinker 1 and sinker 2 are *u* (*m*_1_) = 0.000 021 g and *u* (*m*_2_) = 0.000 023 g, and each uncertainty estimate has 6 − 1 = 5 degrees of freedom (*df_m_*_1_ = *df_m_*_2_ = 5).

The values of *m*_cal_ and *m*_tare_ were estimated in a similar fashion; however, there is only one determination of each mass, and *u* (*m*_cal_) = *u* (*m*_tare_) = 0.000 050 g. Because there is only one observation for *m*_cal_ and *m*_tare_, we will use engineering judgment to determine the degrees of freedom associated with *u* (*m*_cal_) and *u* (*m*_tare_). Assuming that the “uncertainty of the uncertainty” is 50 %, there are two degrees of freedom associated with each uncertainty estimate (*df_m_*_cal_ = *df_m_*_tare_ = 2).

#### 5.5.3 Uncertainties *u* (*V*_cal_) and *u* (*V*_tare_) in Volumes of the Calibration Masses

The limits to error of *V*_cal_ and *V*_tare_ were estimated to be 0.05 % of the nominal sinker volume based on engineering judgment. Assuming the limits represent a uniform distribution, the standard uncertainties associated with *V*_cal_ and *V*_tare_ are

$$u\left(V_{\mathrm{cal}}\right)=\frac{0.0005\cdot V_{\mathrm{cal}}}{\sqrt{3}}\mathrm{and}\;u\left(V_{\mathrm{tare}}\right)=\frac{0.0005\cdot V_{\mathrm{tare}}}{\sqrt{3}}.$$ (37)

We determined that eight degrees of freedom were appropriate based on the assumption that the “uncertainty of the uncertainty” is 25 % (*df_V_*_cal_ = *df_V_*_tare_ = 8).

#### 5.5.4 Uncertainty *u* (*V*_1_) and *u* (*V*_2_) in Sinker Volumes

The determination of the sinker volumes involves the determination of their volumes at 20 °C and atmospheric pressure by the hydrostatic experiment described in Sec. 3.3. These values must then be adjusted for the effects of temperature and pressure. Each of these components involves multiple sources of uncertainties.

##### 5.5.4.1 Uncertainty *u* (*V*_ref_) in Sinker Volumes at 20 °C

A summary of the uncertainties contributing to the sinker volume uncertainty at the reference temperature of 20 °C is presented in Table 7. The uncertainty in the density of the silicon standards is that assigned by the NIST Mass Group [9]. The uncertainty in the mass determinations of the standards and sinkers includes the balance linearity, uncertainty in the calibration masses, uncertainty in air buoyancy, and possible surface adsorption of water. For the hydrostatic weighings, the effects of the balance calibration and linearity are reduced because of the relatively small weight differences measured. Air buoyancy and surface adsorption do not apply. (The sinkers were immersed in the fluid for more than 48 hours prior to the volume determination, giving them time to come to equilibrium with the fluid.) However, the hydrostatic weighings were affected by an observed linear drift of 0.0003 g/h, as determined by the drift in the pan weighings taken every 16 minutes over the course of the test. The largest deviation from the linear trend was 0.000 12 g, with an average of less than 0.000 05 g.

The effects of the error sources on the calculated volumes are given for four cases. The columns labeled “Sinker 1 (Ti)” and “Sinker 2 (Ta)” are for the two sinkers, where the silicon standards were taken as the knowns, i.e., the ratios AB, CB, and BC for the Ta sinker and the ratios AD, DA, DC, and CD for the Ti sinker. The column “Si ref to Si” is for the check measurement comparing one silicon standard to the other (the ratio AC). “Ta ref to Ti” is for the calculation of the tantalum sinker volume taking the titanium sinker volume as the known (the ratio DB). The overall uncertainty varied from 0.000 0052 cm^3^ to 0.000 030 cm^3^, with objects having the highest density (i.e., the smallest volume and buoyancy force) having the highest relative uncertainties.

The measured volumes of the two sinkers at the reference temperature *V*_1ref_ and *V*_2ref_ have uncertainties due to both random and systematic effects. The standard uncertainties associated with random errors, from a least-squares analysis of the hydrostatic data, are *u* (*V*_1R_) = 0.000 0023 cm^3^ and *u* (*V*_2R_) = 0.000 0031 cm^3^. There are six degrees of freedom associated with each estimate (*df_V_*_1R_ = *df_V_*_2R_ = 6). The standard uncertainties associated with systematic calibration effects are *u* (*V*_1S_) = 0.000 102 cm^3^ and *u* (*V*_2S_) = 0.000 050 cm^3^. Assuming that the “uncertainty of the uncertainty” is 25 %, eight degrees of freedom are appropriate for the uncertainties due to systematic effects (*df_V_*_1S_ = *df_V_*_2S_ = 8).

Thus, the combined standard uncertainty of the volume of sinker 1 at reference conditions (20 °C and 0.08 MPa) is

$$u\left(V_{1\mathrm{ref}}\right)={\left[u^{2}\left(V_{1R}\right)+u^{2}\left(V_{1S}\right)\right]}^{0.5},$$ (38)

and the degrees of freedom are given by

$$df_{V1\mathrm{ref}}=\frac{u^{4}\left(V_{1\mathrm{ref}}\right)}{\frac{u^{4}\left(V_{1R}\right)}{df_{V1R}}+\frac{u^{4}\left(V_{1S}\right)}{df_{V1S}}}.$$ (39)

Similar equations were used to determine the combined standard uncertainty and degrees of freedom for the measured volume of sinker 2.

##### 5.5.4.2 Uncertainty in Sinker Volumes *u* (*V*_1_) and *u* (*V*_2_) as a Function of *T* and *p*

The volumes of sinker 1 (Ti) and sinker 2 (Ta) determined by the hydrostatic comparator experiment at 20 °C (*V*_1ref_ and *V*_2ref_) must be modified by three additional corrections to account for temperature and pressure effects:

$$V_{i}=V_{i,\mathrm{ref}}\cdot V_{\kappa }\cdot V_{\alpha }\cdot V_{T},$$ (40)

where *V_κ_* accounts for pressure effects and *V_α_* and *V_T_* account for temperature effects (i.e., thermal expansion).

The combined standard uncertainties for the volumes of the sinkers, based on propagation of errors and independent input quantities, are given by

$$\begin{array}{l} u\left(V_{i}\right)=[{\left(\frac{\partial V_{i}}{\partial V_{i,\mathrm{ref}}}\right)}^{2}u^{2}\left(V_{i,\mathrm{ref}}\right)+{\left(\frac{\partial V_{i}}{\partial V_{\kappa }}\right)}^{2}u^{2}\left(V_{\kappa }\right)\\ \;{+{\left(\frac{\partial V_{i}}{\partial V_{T}}\right)}^{2}u^{2}\left(V_{T}\right)]}^{0.5}. \end{array}$$ (41)

The uncertainty of the temperature correction *V_T_* also includes uncertainty of the *V_α_* correction (as discussed below), so *u* (*V_α_*) does not appear in the uncertainty calculation. The degrees of freedom associated with *u* (*V*_1_) are

$$\begin{array}{l} df_{V1}=\\ \frac{u^{4}\left(V_{1}\right)}{\frac{{\left(\frac{\partial V_{1}}{\partial V_{1\mathrm{ref}}}\right)}^{4}u^{4}\left(V_{1\mathrm{ref}}\right)}{df_{V1\mathrm{ref}}}+\frac{{\left(\frac{\partial V_{1}}{\partial V_{\kappa }}\right)}^{4}u^{4}\left(V_{\kappa }\right)}{df_{V\kappa }}+\frac{{\left(\frac{\partial V_{1}}{\partial V_{T}}\right)}^{4}u^{4}\left(V_{T}\right)}{df_{VT}}}. \end{array}$$ (42)

A similar equation is used to compute *df_V_*2.

The correction, *V_κ_*, is defined as

$$V_{\kappa }=1-\frac{p-p_{\mathrm{ref}}}{\kappa_{0}},$$ (43)

where *κ*_0_ is the bulk modulus of the sinker material. The uncertainties of the two pressures are negligible compared to the uncertainty of *κ*_0_. Thus, the standard uncertainty of *V_κ_* is

$$\begin{array}{l} u\left(V_{\kappa }\right)=\sqrt{{\left(\frac{\partial V_{\kappa }}{\partial \kappa_{0}}\right)}^{2}u^{2}\left(\kappa_{0}\right)}=\sqrt{{\left(\frac{p-p_{\mathrm{ref}}}{\kappa_{0}^{2}}\right)}^{2}u^{2}\left(\kappa_{0}\right)}\\ \;=\left(\frac{p-p_{\mathrm{ref}}}{\kappa_{0}^{2}}\right)u\left(\kappa_{0}\right), \end{array}$$ (44)

where *u* (*κ*_0_) = 0.05 ⋅ *κ*_0_, based on engineering judgment. The value of *κ*_0_ for titanium is 108.4 × 10^6^ GPa^−1^ and the value for tantalum is 196.3 × 10^6^ GPa^−1^ [14]. Assuming that the “uncertainty of the uncertainty” is 25 %, 8 degrees of freedom were appropriate (*df_Vκ_* = 8).

Because volume measurements were taken at a nominal temperature of 20 °C, we need to correct the volume of the sinkers for density measurements taken at other temperatures. *V_α_* is a correction based on measured values of the thermal expansion of the titanium and tantalum used to fabricate the sinkers (see [1]).

**Fig. 10:**
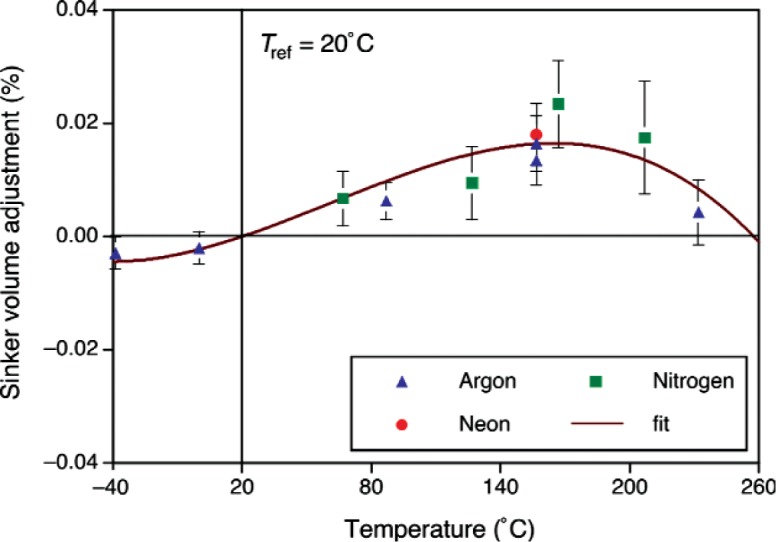
Sinker volume adjustment as a function of temperature based on measurements of low-density gases (adapted from McLinden [15]). The reference temperature for the adjustment is 20.00 °C, and the error bars represent standard uncertainties.

*V_T_* is an additional calibration based on measurements of low-pressure (i.e., nearly ideal) gases in the two-sinker densimeter. Gas densities were measured at several (nearly identical) pressures along several isotherms. The densities at corresponding pressures along pairs of isotherms were ratioed and extrapolated to zero pressure, where the ideal-gas law applies:

$$\frac{\rho \left(T\right)}{\rho \left(T_{\mathrm{ref}}\right)}=\frac{T_{\mathrm{ref}}}{T},$$ (45)

where *T*_ref_ is the temperature (293.15 K) of the hydrostatic sinker volume determination. The basic concept is that of a gas thermometer, except inverted (i.e., the temperatures are the known quantities and the densities are the unknown quantities, rather than vice versa). The difference (in percent) between the extrapolated density ratio and the measured temperature ratio for a given pair of isotherms is the percentage adjustment in the sinker volumes resulting from this calibration. The results are summarized in Fig. 10 for these calibrations on three different gases. (See McLinden [15] for a complete discussion of this calibration, its uncertainties, and a listing of the data.)

We used weighted least-squares regression to fit a cubic polynomial, which was constrained to pass through zero at the reference temperature of 20 °C, to the sinker volume adjustment data. A quadratic equation based on the standard uncertainties given by McLinden [15] (and shown by the error bars in Fig. 10) was used as the weighting function for the regression analysis. The weighted regression equation was used to estimate *V_T_*, and the standard uncertainty of *V_T_* is the standard error of a predicted *V_T_*. Both *V_T_* and *u* (*V_T_*) depend on the temperature; values of *V_T_* range from − 0.0036 % to 0.0163 % of the sinker volume, and values of *u* (*V_T_*) range from 0.0021 % to 0.0105 %. Since 11 observations and three model parameters were used in the fit, there are 8 degrees of freedom associated with *u* (*V_T_*) (*df_VT_* = 8).

The *V_T_* calibration was applied to sinker volume data that include the *V_α_* correction, and any error in *V_α_* will result in a different value for the *V_T_* calibration. Indeed, the entire purpose of the *V_T_* calibration is to improve upon the *V_α_* correction. This is why *u* (*V_α_*) does not appear in Eqs. (41) and (42).

#### 5.5.5 Uncertainty in Purge Gas Density *u* (*ρ*_N2_)

The density of the nitrogen purge gas in the balance chamber was computed with a virial expansion

$$\rho_{N2}=\frac{W_{m}}{2B}\left[-1+\sqrt{1+\frac{4Bp}{RT}}\right],$$ (46)

where *W_m_* is the molar mass, *R* is the molar gas constant, and the second virial coefficient *B* is a function of temperature given by Span et al. [16]. The estimated uncertainty in the nitrogen density calculated by Eq. (46) at the near-ambient conditions of interest here is less than 0.01 %. The combined standard uncertainty of the density of nitrogen, *u* (*ρ*_N2_), is a function of pressure and temperature, so that

$$u\left(\rho_{N2}\right)=\sqrt{{\left(\frac{\partial \rho_{N2}}{\partial T}\right)}^{2}u^{2}\left(T\right)+{\left(\frac{\partial \rho_{N2}}{\partial p}\right)}^{2}u^{2}\left(p\right)},$$ (47)

with degrees of freedom

$$df_{N2}=\frac{u^{4}\left(\rho_{N2}\right)}{\frac{{\left(\frac{\partial \rho_{N2}}{\partial T}\right)}^{4}u^{4}\left(T\right)}{df_{T}}+\frac{{\left(\frac{\partial \rho_{N2}}{\partial p}\right)}^{4}u^{4}\left(p\right)}{df_{p}}}.$$ (48)

We need to determine *u* (*T*), *u* (*p*), *df_T_*, and *df_p_* to calculate *u* (*ρ_N_*_2_) and *df_N_*_2_. Each of the uncertainties *u* (*T*) and *u* (*p*) has a random component since we use the average of six repeat measurements in each density calculation. The uncertainties also have systematic components that are given by the standard uncertainties 0.2 K for temperature and 0.01 % for pressure. The combined standard uncertainties for temperature and pressure are

$$u\left(T\right)={\left[{\left(\frac{1}{\sqrt{6}}S_{T}\right)}^{2}+{\left(0.2K\right)}^{2}\right]}^{0.5}$$ (49)

and

$$u\left(p\right)={\left[{\left(\frac{1}{\sqrt{6}}S_{p}\right)}^{2}+{\left(0.0001\cdot p\right)}^{2}\right]}^{0.5},$$

where *S_T_* and *S_p_* are standard deviations in the six temperature and pressure readings, respectively. There are five degrees of freedom associated with each random component. Assuming the “uncertainty of the uncertainty” of each systematic component is 25 %, based on engineering judgment, there are eight degrees of freedom associated with each systematic component. The degrees of freedom for *u* (*T*) and *u* (*p*) can be computed from the Welch-Satterthwaite approximation, as follows:

$$df_{T}=\frac{u^{4}\left(T\right)}{\frac{1}{5}{\left(\sqrt{\frac{1}{6}}S_{T}\right)}^{4}+\frac{1}{8}{\left(0.2K\right)}^{4}},$$ (50)

$$df_{p}=\frac{u^{4}\left(p\right)}{\frac{1}{5}{\left(\sqrt{\frac{1}{6}}S_{p}\right)}^{4}+\frac{1}{8}{\left(0.0001p\right)}^{4}}.$$ (51)

#### 5.5.6 Uncertainty *u* (*W*_cal_), *u* (*W*_tare_), *u* (*W*_1_), and *u* (*W*_2_) in Weighings

The values of *W*_cal_, *W*_tare_, *W*_1_, and *W*_2_ are all averages of ten measurements, so the estimated standard uncertainties of the four weighings are

$$\begin{array}{l} u\left(W_{\mathrm{cal}}\right)=\frac{S_{W\mathrm{cal}}}{\sqrt{10}},\;u\left(W_{\mathrm{tare}}\right)=\frac{S_{W\mathrm{tare}}}{\sqrt{10}},\\ u\left(W_{1}\right)=\frac{S_{W1}}{\sqrt{10}},\;and\;u\left(W_{2}\right)=\frac{S_{W2}}{\sqrt{10}}. \end{array}$$ (52)

Each uncertainty has 9 degrees of freedom (*df_W_*_cal_ = *df_W_*_tare_ = *df_W_*_1_ = *df_W_*_2_ = 9).

#### 5.5.7 Uncertainty *u* (*ρ*_0_) in Apparatus Zero

Zero pressure (or vacuum) density readings were collected between toluene fillings to provide an indication of the amount of drift in the measurement system over time. The vacuum data were collected in the following sequence:

vacuum data “A”: January 5, 2006
toluene filling #1
vacuum data “B": January 20, 2006
vacuum data “C”: February 1, 2006
toluene fillings #2 and 2a
vacuum data “D”: February 24, 2006
toluene fillings #3, 3a, and 3b
vacuum data “E”: March 11, 2006.

Four separate straight-line regression equations were fit to consecutive pairs of vacuum measurements. For example, a straight line fit to vacuum data A and vacuum data B would be used to estimate the amount of drift for measurements taken during toluene filling #1. Thus, the regression equations depend on the elapsed time from the start of the experiment to the measurement of interest. The estimated density *ρ*_0_ for a measurement taken at elapsed time *τ*_0_ is

$$\rho_{0}=c_{0}+c_{1}\tau_{0},$$ (53)

where *c*_0_ and *c*_1_ are fitting parameters, and the standard uncertainty of *ρ*_0_ is

$$u\left(\rho_{0}\right)=s{\left[\frac{1}{n}+{\left(\tau_{0}-\bar{\tau }\right)}^{2}/\sum_{i=1}^{n}{\left(\tau_{i}-\bar{\tau }\right)}^{2}\right]}^{0.5},$$ (54)

where *n* is the number of observations used to estimate the regression line and *s* is the standard deviation of the fit. There are *n* − 2 degrees of freedom associated with *u* (*ρ*_0_) (*df_ρ_*_0_ = *n* − 2).

#### 5.5.8 Summary of *u* (*ρ*_fluid_)

Table 8 displays two examples of calculations of *u* (*ρ*_fluid_) for density determinations near ambient conditions (*t* = 20 °C and *p* = 1.0 MPa, Table 8a) and for more extreme conditions (*t* = 150 °C and *p* = 30 MPa, Table 8b). Since there are multiple observations at the selected temperature and pressure combinations, the values in the table represent average uncertainties and sensitivity coefficients. The information in the table provides some insight into the role of the magnitude of the uncertainty and sensitivity coefficient for each source of uncertainty. Table 8 considers only the apparatus/method uncertainties and their associated degrees of freedom; they are a subset of the overall combined uncertainty discussed in Sec. 5.7.

Although there are 14 individual sources of uncertainty in *u* (*ρ*_fluid_), not all sources contribute a significant amount to the total uncertainty. We selected a few temperature and pressure combinations and computed the percentage of the total variation for all 14 sources of uncertainty. Table 9 displays the percentage of total variation for the top six contributing sources as well as the combined percentage of the remaining eight sources and the value of *u* (*ρ*_fluid_). Again, the values in the table represent average percentages and the average uncertainties. The largest contributor to *u* (*ρ*_fluid_) for all temperature and pressure combinations is *u* (*V*_1_), the uncertainty in the volume of the titanium sinker, followed by *u* (*V*_2_), the volume of the tantalum sinker. These two sources of uncertainty account for 98 % to 99 % of the total variation in *ρ*_fluid_.

### 5.6 Uncertainty in Temperature and Pressure *u (tp*)

Since the fluid density is a function of temperature and pressure, uncertainties in the measured temperature and pressure will contribute to the uncertainty of the reported density. A sensitivity study was used to estimate *u* (*tp*) in which temperature and pressure were varied in Eq. (10) according to their corresponding uncertainty levels. The uncertainty *u* (*tp*) was determined from the resulting density values. For the present measurements *u* (*t*) = 0.002 °C and *u* (*p*) = 2 kPa, so that *u* (*tp*) = 0.0025 kg/m^3^. Assuming that the “uncertainty of the uncertainty” is 25 %, eight degrees of freedom are appropriate for this uncertainty (*df_tp_* = 8). The very small magnitude of this effect is a result of the nearly-incompressible nature of toluene over the temperatures and pressures studied. This effect would be much more significant if the present apparatus were used for measurements on a gas or a fluid near its critical point.

When this SRM is used in the calibration of a densimeter, *u* (*tp*) depends on the user’s temperature and pressure errors. Since each user’s measurement apparatus is different, we performed a sensitivity study for this uncertainty by varying temperature and pressure according to nine different combinations of error levels and quantifying the effect on density. The results of the sensitivity study are shown in Table 10. The error values listed in the first two columns of Table 10 represent a user’s limits to error (rather than a standard uncertainty), and the values of *u* (*tp*) are typical uncertainties across all temperatures and pressures in the test region. Ultimately, the user is responsible for estimating an appropriate value of *u* (*tp*) and its associated degrees of freedom.

### 5.7 Combined Standard Uncertainty

The combined standard uncertainty associated with an estimated fluid density is given by Eq. (17). The values of *u* (*ρ*), *u* (*V*), and *u* (*x*) are constant for all temperature and pressure combinations. The value of *u* (*e*) depends on the operating temperature and pressure and is calculated from Eq. (24). The value of *u* (Δ) depends on the degree of air saturation in the measured sample (for degassed samples, *u* (Δ) = 0), and *u* (*tp*) depends on the level of error associated with the operating temperature and pressure in the user’s apparatus.

The values of the individual uncertainty components for the measurements described in this work are displayed along with their associated degrees of freedom in Table 11. Table 12 displays the combined standard uncertainty for four of the uncertainty components, *u* (*ρ*), *u* (*V*), *u* (*x*) and *u* (*e*) (from Table 11 and Eq. 24) for the same even increments of temperatures and pressures listed in Table 4.

### 5.8 Expanded Uncertainty and Degrees of Freedom

The expanded uncertainty is *U* = *ku_C_*, where the coverage factor *k* is obtained from the Student’s *t* distribution based on the effective degrees of freedom for *u_C_*. In general, the expanded uncertainty associated with a 100 · (1 − *α*) % coverage probability (*α* is 0.05 for 95 % coverage) is given by $U=t_{\left(1-\alpha /2,df_{e\mathrm{ff}}\right)}\cdot u_{C}$. Typically, *k* = 2 is used to compute the expanded uncertainty associated with a 95 % uncertainty interval. However, if the effective degrees of freedom are less than 30, the interval coverage is less than 95 %. Thus, we recommend that the effective degrees of freedom be computed to determine the proper coverage factor.

The effective degrees of freedom obtained from the Welch-Satterthwaite approximation are

$$df_{\mathrm{eff}}=\frac{u_{C}^{4}}{\frac{u^{4}\left(\rho \right)}{df_{\rho }}+\frac{u^{4}\left(V\right)}{df_{V}}+\frac{u^{4}\left(x\right)}{df_{x}}+\frac{u^{4}\left(e\right)}{df_{e}}+\frac{u^{4}\left(\Delta \right)}{df_{\Delta }}+\frac{u^{4}\left(tp\right)}{df_{tp}}}.$$ (55)

Since the effective degrees of freedom depend on the value of *u* (*e*) for a given temperature and pressure, we will provide a conservative estimate of *df*_eff_ for all degassed measurements given in this document. The smallest (most conservative) value of *df*_eff_ is observed when *u* (*e*) is large since all other uncertainty sources and degrees of freedom are fixed. We used the largest value of *u* (*e*) observed for our data, *u* (*e*) = 0.053 kg/m^3^ (*df_e_* = 9), and the remaining uncertainties and associated degrees of freedom from Table 11 to compute *df*_eff_. Thus, a conservative value for all degassed measurements reported in this document is *df*_eff_ = 10, and the appropriate coverage factor for a 95 % uncertainty interval is 2.228.

When the SRM is used to calibrate a user’s densimeter, two of the uncertainty components of *u_C_*, ie. *u* (Δ) and *u* (*tp*), depend on the user’s conditions, so the final value of the user’s *df*_eff_ cannot be calculated in this document.

### 5.9 Uncertainty Example

We shall estimate the density and its uncertainty at *t* = −23 °C and *p* = 12 MPa for a degassed sample (Δ = 0 kg/m^3^ and *u* (Δ) = 0 kg/m^3^). The values of temperature and pressure are input into Eq. (10), resulting in a density estimate of 913.461 kg/m^3^. Similarly, *u* (*e*) = 0.0292 kg/m^3^ is calculated from Eq. (24). If the limit to temperature error is thought to be ± 0.1 °C, and the limit to pressure error is ± 0.1 MPa, then from Table 10, *u* (*tp*) = 0.075 kg/m^3^. The combined standard uncertainty of the estimated density is then

$$\begin{array}{l} u_{C}={\left[u^{2}\left(\rho \right)+u^{2}\left(V\right)+u^{2}\left(x\right)+u^{2}\left(e\right)+u^{2}\left(tp\right)\right]}^{0.5}\\ u_{C}={\left[\begin{array}{l} {\left(0.0086\mathrm{kg}/m^{3}\right)}^{2}+{\left(0.0114\mathrm{kg}/m^{3}\right)}^{2}\\ +{\left(0.003\mathrm{kg}/m^{3}\right)}^{2}+{\left(0.0292\mathrm{kg}/m^{3}\right)}^{2}\\ +{\left(0.075\mathrm{kg}/m^{3}\right)}^{2} \end{array}\right]}^{0.5}\\ u_{C}=0.082\mathrm{kg}/m^{3}. \end{array}$$ (56)

If the levels of error defining *u* (*tp*) are well known, then we will assume *df_tp_* = 30. Using *df_e_* = 9 from Sec. 5.5, the effective degrees of freedom associated with *u C* are

$$\begin{array}{l} df_{\mathrm{eff}}=\frac{u_{C}^{4}}{\frac{u^{4}\left(\rho \right)}{df_{\rho }}+\frac{u^{4}\left(V\right)}{df_{V}}+\frac{u^{4}\left(x\right)}{df_{x}}+\frac{u^{4}\left(e\right)}{df_{e}}+\frac{u^{4}\left(tp\right)}{df_{tp}}}\\ df_{\mathrm{eff}}={\left(0.082\mathrm{kg}/m^{3}\right)}^{4}/\\ \;[\frac{{\left(0.0086\mathrm{kg}/m^{3}\right)}^{4}}{886}+\frac{{\left(0.0114\mathrm{kg}/m^{3}\right)}^{4}}{32}\\ \;+\frac{{\left(0.003\mathrm{kg}/m^{3}\right)}^{4}}{8}+\frac{{\left(0.0292\mathrm{kg}/m^{3}\right)}^{4}}{9}\\ \;+\frac{{\left(0.075\mathrm{kg}/m^{3}\right)}^{4}}{30}]\\ df_{\mathrm{eff}}=39, \end{array}$$ (57)

and the appropriate coverage factor for a 95 % uncertainty interval is 2.0227.

## 6. Discussion and Conclusions

We report values for the density of liquid toluene that form the basis of NIST Standard Reference Material^®^ 211d “Toluene Liquid Density—Extended Range.” This work extends the range of this SRM, which was previously limited to 15 °C to 25 °C and normal atmospheric pressure, to the temperature range −50 °C to 150 °C and pressure range 0.1 MPa to 30 MPa. This SRM will be invaluable in calibration of industrial densimeters.

The uncertainties for the density values were obtained by a thorough statistical analysis of multiple sources of uncertainty. In many cases, a measured quantity depends on other underlying measurands, and the uncertainties at each level were considered. We have presented the uncertainty analysis in considerable detail with the hope that it will serve as an example for others carrying out fluid property measurements.

The measurements reported here are directly traceable to SI quantities. The density was determined by weighing sinkers immersed in the fluid. The volume (or, equivalently, density) of the sinkers was determined by comparison to solid density standards that are directly traceable to the meter and kilogram. The balance that carried out the weighings was calibrated for each density determination using calibration weights, which were, in turn, calibrated against standard masses. The temperature of the fluid was measured with a standard platinum resistance thermometer calibrated with ITS–90 fixed points. The pressure transducer was calibrated against a piston gage pressure standard.

## Figures and Tables

**Table 1:** Volume ratios and volumes determined by hydrostatic weighing

Object	Ratio	Measured Volume Ratio	Ratio Adjusted to 20 °C	Mass (g)	Volume (cm^3^)
tantalum sinker	AB	11.746 127	11.746 129		3.610 246
	CB	11.774 715	11.774 718		3.610 248
	BC	0.084 928	0.084 928		3.610 242
	average			60.177 96	3.610 245
					*σ* = 0.000 003
titanium sinker	AD	3.177 097	3.177 098		13.347 530
	DA	0.314 753	0.314 753		13.347 530
	DC	0.313 989	0.313 989		13.347 572
	CD	3.184 823	3.184 824		13.347 566
	average			60.163 41	13.347 549
					*σ* = 0.000 023

**Table 2:** Volume ratios determined by hydrostatic weighing compared to calculated values

Ratio	Measured Value	Calculated Value	Difference
AC	0.997 576	0.997 571	0.000 005
DB	3.697 088	3.697 131	−0.000 043

**Table 3:** Parameters for empirical model (Eq. 10)

*k*	*a_k_*	*b_k_*	*c_k_*
1	0.118 648 × 10^4^	0	0
2	−0.133 648 × 10^3^	−0.80	0
3	−0.119 260 × 10^−1^	−5.34	0
4	0.229 402	−0.10	1.00
5	0.187 212 × 10^−4^	−7.60	1.00
6	0.661 127 × 10^−1^	−2.20	1.15
7	−0.249 953 × 10^−1^	−2.24	1.30
8	−0.280 091 × 10^−5^	−7.93	1.30

**Table 4:** Estimated fluid density *ρ* in kg/m^3^ for degassed samples (g = 0 kg/m^3^) calculated from Eq. (10)

*t* (°C)	0.1	Pressure (MPa)							
		1	2	5	10	15	20	25	30
−50	931.655	932.100	932.605	934.118	936.595	939.011	941.370	943.678	945.937
−40	922.362	922.833	923.366	924.963	927.573	930.114	932.592	935.012	937.377
−30	913.101	913.598	914.162	915.848	918.600	921.274	923.877	926.415	928.893
−20	903.860	904.386	904.982	906.763	909.665	912.481	915.216	917.879	920.475
−10	894.627	895.184	895.815	897.699	900.762	903.726	906.602	909.397	912.118
0	885.392	885.982	886.651	888.645	891.878	895.002	898.027	900.962	903.813
10	876.142	876.769	877.478	879.589	883.006	886.300	889.482	892.564	895.554
20	866.864	867.531	868.284	870.522	874.136	877.610	880.960	884.198	887.334
30	857.545	858.255	859.056	861.432	865.257	868.924	872.453	875.856	879.145
40	848.170	848.929	849.782	852.307	856.359	860.233	863.952	867.530	870.982
50	838.726	839.537	840.448	843.134	847.432	851.529	855.450	859.215	862.838
60	829.195	830.065	831.038	833.902	838.466	842.802	846.939	850.902	854.706
70	819.562	820.496	821.539	824.597	829.450	834.043	838.413	842.586	846.581
80	809.808	810.815	811.935	815.205	820.373	825.244	829.863	834.260	838.458
90	799.916	801.003	802.208	805.713	811.225	816.397	821.283	825.919	830.330
100	789.865	791.043	792.342	796.106	801.994	807.492	812.665	817.556	822.194
110	779.634	780.914	782.318	786.369	792.669	798.521	804.005	809.167	814.044
120	*	770.596	772.118	776.486	783.239	789.478	795.295	800.748	805.878
130	*	760.069	761.722	766.443	773.694	780.353	786.531	792.294	797.691
140	*	749.309	751.110	756.222	764.022	771.140	777.707	783.803	789.481
150	*	738.293	740.259	745.808	754.214	761.832	768.820	775.270	781.246

* above the normal boiling point temperature (liquid phase not stable at *p* = 0.1 MPa)

**Table 5:** Uncertainty and degrees of freedom associated with vial-to-vial variability at near-ambient conditions (from Bean and Houser [4])

Source	Uncertainty (kg/m^3^)	Degrees of Freedom
Apparatus	*u* (*A*) = 0.0032	*df_A_* = ∞
Day-to-day	*u* (*D*) = 0.0047	*df_D_* = 5
Ampoule-to-ampoule	*u* (*v*) = 0.0099	*df_v_* = 23

Total	*u* (*V*) = 0.0114	*df_V_* = 32

**Table 6:** Calibration data for the standard masses used in this work

Nominal Mass (g)	True Mass (g)	Uncertainty (g)	Density (g/cm^3^)
50	50.000 1507	0.000 011 45	7.85
10	9.999 9966	0.000 008 15	7.85
2	2.000 0193	0.000 0032	7.85

**Table 7:** Summary of standard uncertainties in volumes determined by hydrostatic weighing

Source of Error	Magnitude of Error	Sinker 1 (Ti)	Uncertainty in Volume (cm^3^)		Ta ref to Ti
			Sinker 2 (Ta)	Si ref to Si	
Density of standard	1.6 × 10^−5^ g/cm^3^	9.15 × 10^−5^	2.48 × 10^−5^	29.2 × 10^−5^	2.66 × 10^−5^
Mass of standard	5.0 × 10^−5^ g	0.29 × 10^−5^	0.80 × 10^−5^	0.92 × 10^−5^	0.53 × 10^−5^
Mass of object	5.0 × 10^−5^ g	3.07 × 10^−5^	3.07 × 10^−5^	3.07 × 10^−5^	3.07 × 10^−5^
Weighing of standard	5.0 × 10^−5^ g	0.96 × 10^−5^	0.26 × 10^−5^	3.07 × 10^−5^	0.83 × 10^−5^
Weighing of object	5.0 × 10^−5^ g	3.07 × 10^−5^	3.07 × 10^−5^	3.07 × 10^−5^	3.07 × 10^−5^

Root-sum-of-squares		10.2 × 10^−5^	5.07 × 10^−5^	29.7 × 10^−5^	5.19 × 10^−5^

**Table 8a:** Uncertainty “budget” for *ρ* fluid (Eq. 26) for two sets (a, b) of operating conditions. The sensitivity coefficients have been multiplied by 1000 to convert to kg/m^3^

Source	*t* = 20 °C, *p* = 1 MPa			
	Standard Uncertainty	Sensitivity Coefficient	*c_i_* ⋅ *u* (*x_i_*) (kg/m^3^)	Degrees of Freedom
	*u* (*x_i_*)	*c_i_*		
*W*_cal_	2.557 × 10^−7^ g	42.039	1.075 × 10^−5^	9
*W*_tare_	2.131 × 10^−7^ g	56.478	1.203 × 10^−5^	9
*m*_cal_	5.000 × 10^−5^ g	42.041	2.102 × 10^−3^	2
*m*_tare_	5.000 × 10^−5^ g	56.481	2.824 × 10^−3^	2
*V*_cal_	2.159 × 10^−3^ cm^3^	0.039	8.362 × 10^−5^	8
*V*_tare_	2.159 × 10^−3^ cm^3^	0.053	1.142 × 10^−4^	8
*ρ*_air_	6.295 × 10^−7^ g/cm^3^	107.996	6.799 × 10^−5^	8
*m*_1_	2.100 10^−5^ × g	97.345	2.044 × 10^−3^	5
*m*_2_	2.300 × 10^−5^ g	82.905	1.907 × 10^−3^	5
*V*_1_	3.161 × 10^−4^ g	84.451	2.670 × 10^−2^	9
*V*_2_	9.513 × 10^−5^ g	71.924	6.842 × 10^−3^	13
*W*_1_	1.832 × 10^−6^ g	97.342	1.784 × 10^−4^	9
*W*_2_	3.967 × 10^−7^ g	82.903	3.289 × 10^−5^	9
*ρ*_0_	4.402 × 10^−9^ 3 g/cm	1000.0	4.402 × 10^−6^	48
$\begin{array}{c} u(\rho_{\text{fluid}})={\left[\sum {\left(c_{i}\cdot u(x_{i})\right)}^{2}\right]}^{0.5}=0.027\;\text{kg}/m^{3}\\ df_{\text{eff}}=11 \end{array}$				

**Table 8b:** 

Source	*t* = 150 °C, *p* = 30 MPa			
	Standard Uncertainty	Sensitivity Coefficient	*c_i_* ⋅ *u* (*x_i_*) kg/m^3^	Degrees of Freedom
	*u* (*x_i_*)	*c_i_*		
*W*_cal_	1.281 × 10^−6^ g	37.860	4.851 × 10^−5^	9
*W*_tare_	4.202 × 10^−7^ g	50.865	2.138 × 10^−5^	9
*m*_cal_	5.000 × 10^−5^ g	37.862	1.893 × 10^−3^	2
*m*_tare_	5.000 × 10^−5^ g	50.867	2.543 × 10^−3^	2
*V*_cal_	2.159 × 10^−3^ cm^3^	0.036	7.784 × 10^−5^	8
*V*_tare_	2.159 × 10^−3^ cm^3^	0.048	1.035 × 10^−4^	8
*ρ*_air_	6.416 × 10^−7^ g/cm^3^	97.261	6.240 × 10^−5^	8
*m*_1_	2.100 × 10^−5^ g	97.532	2.048 × 10^−3^	5
*m*_2_	2.300 × 10^−5^ g	84.527	1.944 × 10^−3^	5
*V*_1_	5.904 × 10^−4^ cm^3^	76.189	4.498 × 10^−2^	10
*V*_2_	1.596 × 10^−4^ cm^3^	66.030	1.054 × 10^−2^	10
*W*_1_	1.019 × 10^−5^ cm^3^	97.530	9.941 × 10^−4^	9
*W*_2_	5.222 × 10^−6^ cm^3^	84.526	4.414 × 10^−4^	9
*ρ*_0_	3.226 × 10^−9^ g/cm^3^	1000.0	3.226 × 10^−6^	38
$\begin{array}{c} u_{c}={\left[\sum {\left(c_{i}\cdot u(x_{i})\right)}^{2}\right]}^{0.5}=0.046\;\text{kg}/m^{3}\\ df_{\text{eff}}=11 \end{array}$				

**Table 9:** Percentages of total variation in *u* (*ρ*_fluid_) for six sources of uncertainty at various temperatures and pressures. The column labeled “all others” contains the combined percentage of total variation for the remaining eight sources. The value of *u* (*ρ*_fluid_) is also listed. The quantities in the table represent average values for the given temperatures and pressures

*t* (°C)	*p* (MPa)	Percent of Total Variation							*u* (*ρ*_fluid_) (kg/m^3^)
		*V*_1_	*V*_2_	*m*_cal_	*m*_tare_	*m*_1_	*m*_2_	all others	
−50	1	92.7	5.6	0.4	0.7	0.3	0.3	0.0	0.036
−50	15	93.0	5.4	0.4	0.7	0.3	0.3	0.0	0.037
0	1	91.0	6.1	0.6	1.2	0.6	0.5	0.0	0.027
0	15	91.7	5.7	0.6	1.1	0.5	0.4	0.0	0.028
50	1	92.1	5.8	0.4	0.8	0.4	0.4	0.0	0.031
50	15	92.5	5.6	0.4	0.7	0.4	0.4	0.0	0.032
50	30	93.4	5.0	0.4	0.6	0.3	0.3	0.0	0.035
100	1	93.0	5.7	0.3	0.5	0.3	0.3	0.0	0.037
100	15	93.3	5.5	0.3	0.5	0.3	0.2	0.0	0.039
150	1	93.4	5.6	0.2	0.3	0.2	0.2	0.0	0.042
150	15	93.6	5.5	0.2	0.3	0.2	0.2	0.0	0.044
150	30	93.9	5.2	0.2	0.3	0.2	0.2	0.1	0.046

**Table 10:** Estimated uncertainty *u* (*tp*) due to user’s temperature and pressure uncertainties

Limit to Temperature Error (°C)	Limit to Pressure Error (MPa)	*u* (*tp*) (kg/m^3^)
± 0.001	± 0.001	0.001
	± 0.01	0.005
	± 0.1	0.051
± 0.01	± 0.001	0.005
	± 0.01	0.007
	± 0.1	0.051
± 0.1	± 0.001	0.053
	± 0.01	0.054
	± 0.1	0.075

**Table 11:** Uncertainties and degrees of freedom for measurements described in this document *

Source	Uncertainty (kg/m^3^)	Degrees of Freedom
*u* (*ρ*)	0.0086	886
*u* (*V*)	0.0114	32
*u* (*x*)	0.003	8
*u* (*tp*)	0.002 47	8

* Degassed samples only (*u* (Δ) = 0).

**Table 12:** Combined standard uncertainty *u* in kg/m^3^, including the effects of *u* (*ρ*), *u* (*V*), and *u* (*x*) and *u* (*e*)

*t* (°C)	0.1	Pressure (MPa)							
		1	2	5	10	15	20	25	30
−50	0.038	0.038	0.038	0.039	0.039	0.040	0.040	0.041	0.042
−40	0.035	0.035	0.035	0.035	0.036	0.036	0.037	0.038	0.039
−30	0.033	0.033	0.033	0.033	0.034	0.034	0.035	0.036	0.037
−20	0.031	0.031	0.031	0.032	0.032	0.033	0.033	0.034	0.035
−10	0.031	0.031	0.031	0.031	0.031	0.032	0.033	0.034	0.035
0	0.030	0.030	0.031	0.031	0.031	0.032	0.032	0.033	0.034
10	0.031	0.031	0.031	0.031	0.031	0.032	0.033	0.034	0.035
20	0.031	0.031	0.031	0.031	0.032	0.033	0.033	0.034	0.035
30	0.032	0.032	0.032	0.032	0.033	0.033	0.034	0.035	0.036
40	0.033	0.033	0.033	0.033	0.034	0.034	0.035	0.036	0.037
50	0.034	0.034	0.034	0.034	0.035	0.036	0.036	0.037	0.038
60	0.035	0.035	0.035	0.036	0.036	0.037	0.037	0.038	0.039
70	0.037	0.037	0.037	0.037	0.037	0.038	0.039	0.040	0.041
80	0.038	0.038	0.038	0.038	0.039	0.039	0.040	0.041	0.042
90	0.039	0.039	0.039	0.039	0.040	0.040	0.041	0.042	0.043
100	0.040	0.040	0.040	0.040	0.041	0.041	0.042	0.043	0.044
110	0.041	0.041	0.041	0.041	0.042	0.042	0.043	0.044	0.045
120	*	0.042	0.042	0.042	0.043	0.043	0.044	0.045	0.046
130	*	0.043	0.043	0.043	0.044	0.044	0.045	0.046	0.047
140	*	0.044	0.044	0.044	0.044	0.045	0.046	0.047	0.048
150	*	0.044	0.045	0.045	0.045	0.046	0.047	0.048	0.049

* above the normal boiling point temperature (liquid phase not stable at *p* = 0.1 MPa)

## 7. Appendix A–Experimental Values of Density

**Table A1:** Experimentally measured temperatures *t*, pressures *p*, and densities *ρ*_exp_ for degassed SRM toluene with the standard uncertainty *u* (*ρ*_fluid_)

	*t* (°C)	*p* (MPa)	*ρ*_exp_ (kg m^−3^)	*u* (*ρ*_fluid_) (kg m^−3^)
Filling 1				
	−59.999	0.0089	940.943	0.042
	−59.998	0.0090	940.942	0.042
	−59.999	0.0090	940.943	0.042
	−59.998	0.0089	940.943	0.042
	−59.998	0.0088	940.941	0.042
	−59.999	3.8239	942.780	0.042
	−59.999	3.8232	942.781	0.042
	−59.998	3.8266	942.782	0.042
	−59.999	3.8248	942.782	0.042
	−59.999	3.8249	942.781	0.042
	−49.997	19.9442	941.344	0.038
	−49.997	19.9435	941.343	0.038
	−49.998	19.9420	941.342	0.038
	−49.997	19.9431	941.343	0.038
	−49.996	19.9449	941.344	0.038
	−39.997	35.6032	939.966	0.038
	−39.998	35.5999	939.964	0.038
	−39.998	35.6019	939.966	0.038
	−39.997	35.6009	939.965	0.038
	−39.997	35.6034	939.967	0.038
	−39.997	30.2614	937.496	0.036
	−39.996	30.2627	937.496	0.036
	−39.996	30.2633	937.496	0.036
	−39.997	30.2634	937.497	0.036
	−39.996	30.2643	937.497	0.036
	−39.998	25.2278	935.115	0.035
	−39.997	25.2297	935.116	0.035
	−39.998	25.2305	935.117	0.035
	−39.997	25.2323	935.116	0.035
	−39.997	25.2318	935.117	0.035
	−39.998	20.1557	932.661	0.034
	−39.997	20.1571	932.660	0.034
	−39.998	20.1612	932.662	0.034
	−39.996	20.1635	932.663	0.034
	−39.996	20.1602	932.661	0.034
	−39.997	15.2720	930.241	0.033
	−39.997	15.2718	930.241	0.033
	−39.997	15.2726	930.240	0.033
	−39.996	15.2724	930.240	0.033
	−39.997	15.2716	930.240	0.033
	−39.998	10.3463	927.742	0.032
	−39.997	10.3466	927.741	0.032
	−39.996	10.3486	927.742	0.032
	−39.996	10.3489	927.742	0.032
	−39.997	10.3464	927.742	0.032
	−39.997	5.2108	925.070	0.032
	−39.996	5.2123	925.071	0.032
	−39.997	5.2134	925.070	0.032
	−39.997	5.2121	925.071	0.032
	−39.996	5.2137	925.070	0.032
	−39.997	1.0800	922.867	0.032
	−39.997	1.0753	922.865	0.032
	−39.996	1.0756	922.864	0.032
	−39.996	1.0761	922.863	0.032
	−39.997	1.0727	922.862	0.032
	−30.002	15.4119	921.486	0.030
	−30.001	15.4118	921.482	0.030
	−30.001	15.4149	921.484	0.030
	−30.002	15.4134	921.483	0.030
	−30.001	15.4134	921.484	0.030
	−20.002	29.4361	920.184	0.032
	−20.002	29.4313	920.182	0.032
	−20.001	29.4308	920.181	0.032
	−20.000	29.4330	920.182	0.032
	−20.001	29.4324	920.180	0.032
	−20.002	24.6818	917.708	0.031
	−20.001	24.6767	917.706	0.031
	−20.001	24.6755	917.705	0.031
	−20.000	24.6765	917.704	0.031
	−20.000	24.6692	917.700	0.031
	−20.000	20.0503	915.236	0.030
	−20.000	20.0500	915.237	0.030
	−20.000	20.0496	915.236	0.030
	−20.000	20.0494	915.236	0.030
	−19.999	20.0503	915.236	0.030
	−20.000	15.2040	912.587	0.029
	−20.000	15.2049	912.588	0.029
	−19.999	15.2051	912.586	0.029
	−19.999	15.2058	912.587	0.029
	−20.000	15.2062	912.587	0.029
	−20.001	10.1332	909.738	0.028
	−20.001	10.1346	909.737	0.028
	−20.001	10.1322	909.737	0.028
	−20.001	10.1318	909.737	0.028
	−20.001	10.1308	909.735	0.028
	−20.002	5.3089	906.948	0.028
	−20.001	5.3142	906.951	0.028
	−20.001	5.3162	906.950	0.028
	−20.000	5.3166	906.950	0.028
	−20.001	5.3176	906.951	0.028
	−19.999	1.0111	904.389	0.027
	−20.000	1.0099	904.389	0.027
	−20.000	1.0110	904.389	0.027
	−19.998	1.0108	904.388	0.027
	−19.999	1.0101	904.388	0.027
	−10.001	13.9588	903.114	0.028
	−9.999	13.9669	903.117	0.028
	−9.999	13.9669	903.118	0.028
	−9.999	13.9678	903.117	0.028
	−9.998	13.9684	903.117	0.028
	−0.002	26.6266	901.902	0.031
	−0.001	26.6226	901.898	0.031
	0.000	26.6236	901.897	0.031
	0.001	26.6235	901.897	0.031
	0.000	26.6221	901.896	0.031
	0.001	25.1503	901.048	0.030
	0.001	25.1507	901.049	0.030
	0.001	25.1516	901.049	0.030
	0.002	25.1522	901.049	0.030
	0.002	25.1526	901.049	0.030
	0.000	20.0628	898.064	0.029
	0.002	20.0638	898.064	0.029
	0.002	20.0633	898.063	0.029
	0.001	20.0633	898.064	0.029
	0.001	20.0646	898.064	0.029
	0.001	15.1485	895.092	0.028
	0.002	15.1487	895.091	0.028
	0.002	15.1499	895.093	0.028
	0.002	15.1490	895.091	0.028
	0.002	15.1486	895.091	0.028
	0.001	10.0567	891.915	0.028
	0.002	10.0575	891.914	0.028
	0.002	10.0570	891.914	0.028
	0.001	10.0523	891.912	0.028
	0.002	10.0518	891.911	0.028
	0.002	5.0765	888.701	0.027
	0.001	5.0753	888.701	0.027
	0.002	5.0757	888.700	0.027
	0.002	5.0770	888.700	0.027
	0.001	5.0764	888.700	0.027
	0.001	1.1818	886.109	0.027
	0.002	1.1821	886.108	0.027
	0.002	1.1821	886.107	0.027
	0.001	1.1809	886.107	0.027
	0.001	1.1816	886.108	0.027
	10.001	12.8459	884.896	0.028
	10.002	12.8471	884.896	0.028
	10.001	12.8456	884.896	0.028
	10.001	12.8448	884.895	0.028
	10.001	12.8447	884.895	0.028
	20.000	24.2835	883.746	0.031
	20.000	24.2826	883.746	0.031
	20.000	24.2828	883.744	0.031
	20.002	24.2842	883.744	0.031
	20.001	24.2837	883.745	0.031
	20.001	19.9622	880.938	0.030
	20.001	19.9604	880.937	0.030
	20.002	19.9599	880.937	0.030
	20.002	19.9589	880.935	0.030
	20.001	19.9584	880.935	0.030
	20.000	14.9999	877.613	0.029
	20.000	14.9992	877.613	0.029
	20.002	15.0003	877.612	0.029
	20.002	14.9987	877.611	0.029
	20.001	14.9977	877.612	0.029
	20.001	9.9660	874.116	0.029
	20.001	9.9670	874.115	0.029
	20.002	9.9683	874.115	0.029
	20.002	9.9685	874.116	0.029
	20.001	9.9684	874.117	0.029
	20.002	4.9073	870.461	0.028
	20.001	4.9095	870.464	0.028
	20.002	4.9117	870.464	0.028
	20.003	4.9122	870.464	0.028
	20.002	4.9109	870.463	0.028
	20.002	1.0214	867.551	0.028
	20.002	1.0206	867.550	0.028
	20.003	1.0222	867.551	0.028
	20.004	1.0221	867.551	0.028
	20.003	1.0216	867.551	0.028
	30.001	11.5320	866.399	0.030
	30.001	11.5308	866.399	0.030
	30.001	11.5314	866.399	0.030
	30.002	11.5311	866.399	0.030
	30.001	11.5295	866.396	0.030
	40.002	21.8779	865.315	0.032
	40.001	21.8766	865.317	0.032
	40.001	21.8769	865.315	0.032
	40.001	21.8769	865.316	0.032
	40.001	21.8764	865.316	0.032
	50.001	32.0188	864.264	0.036
	50.000	32.0181	864.264	0.036
	50.000	32.0184	864.265	0.036
	50.001	32.0204	864.265	0.036
	50.001	32.0199	864.264	0.036
	50.001	29.8506	862.733	0.035
	50.002	29.8515	862.734	0.035
	50.001	29.8513	862.735	0.035
	50.000	29.8499	862.733	0.035
	50.001	29.8505	862.733	0.035
	50.000	24.8786	859.129	0.034
	50.001	24.8789	859.129	0.034
	50.001	24.8781	859.127	0.034
	50.000	24.8753	859.127	0.034
	50.000	24.8779	859.128	0.034
	50.000	19.9578	855.422	0.033
	50.000	19.9592	855.424	0.033
	50.000	19.9596	855.424	0.033
	50.001	19.9606	855.424	0.033
	50.001	19.9599	855.423	0.033
	50.000	15.0142	851.543	0.032
	50.000	15.0153	851.544	0.032
	50.001	15.0157	851.543	0.032
	50.001	15.0159	851.544	0.032
	50.000	15.0151	851.544	0.032
	50.000	9.9887	847.424	0.032
	50.000	9.9850	847.420	0.032
	50.000	9.9867	847.421	0.032
	50.001	9.9864	847.420	0.032
	50.000	9.9856	847.420	0.032
	50.001	5.1148	843.235	0.031
	50.000	5.1143	843.236	0.031
	50.000	5.1141	843.235	0.031
	50.002	5.1142	843.234	0.031
	50.002	5.1149	843.235	0.031
	50.000	1.0621	839.595	0.031
	50.001	1.0604	839.592	0.031
	50.001	1.0619	839.594	0.031
	50.001	1.0612	839.594	0.031
	50.000	1.0610	839.594	0.031
Filling 2				
	−49.994	22.1054	942.346	0.038
	−49.995	22.1045	942.346	0.038
	−49.996	22.0986	942.344	0.038
	−49.995	22.0998	942.343	0.038
	−49.994	22.0994	942.344	0.038
	−49.995	19.6460	941.208	0.038
	−49.995	19.6488	941.210	0.038
	−49.995	19.6478	941.202	0.038
	−49.995	19.6467	941.201	0.038
	−49.995	19.6465	941.202	0.038
	−49.996	17.4593	940.175	0.037
	−49.996	17.4620	940.177	0.037
	−49.996	17.4623	940.178	0.037
	−49.996	17.4626	940.176	0.037
	−49.995	17.4652	940.177	0.037
	−49.996	15.0378	939.024	0.037
	−49.995	15.0403	939.026	0.037
	−49.996	15.0392	939.025	0.037
	−49.996	15.0385	939.025	0.037
	−49.997	12.1742	937.649	0.037
	−49.996	12.1742	937.649	0.037
	−49.995	12.1770	937.649	0.037
	−49.996	12.1760	937.649	0.037
	−49.996	12.1774	937.651	0.037
	−49.997	10.0824	936.632	0.036
	−49.996	10.0842	936.632	0.036
	−49.996	10.0839	936.631	0.036
	−49.997	10.0826	936.632	0.036
	−49.996	10.0848	936.632	0.036
	−49.996	7.9616	935.590	0.036
	−49.995	7.9614	935.590	0.036
	−49.995	7.9623	935.590	0.036
	−49.996	7.9603	935.589	0.036
	−49.996	7.9593	935.588	0.036
	−49.996	6.0743	934.654	0.036
	−49.996	6.0761	934.654	0.036
	−49.996	6.0749	934.654	0.036
	−49.997	6.0732	934.655	0.036
	−49.995	6.0759	934.655	0.036
	−49.999	4.0086	933.623	0.036
	−49.996	4.0198	933.626	0.036
	−49.996	4.0193	933.624	0.036
	−49.996	4.0191	933.625	0.036
	−49.996	4.0209	933.626	0.036
	−49.997	2.1715	932.692	0.036
	−49.995	2.1737	932.691	0.036
	−49.996	2.1744	932.693	0.036
	−49.996	2.1733	932.692	0.036
	−49.996	2.1740	932.693	0.036
	−49.996	0.7106	931.948	0.036
	−49.996	0.7105	931.946	0.036
	−49.996	0.7136	931.948	0.036
	−49.996	0.7132	931.948	0.036
	−49.995	0.7160	931.949	0.036
	−39.996	15.8462	930.527	0.033
	−39.996	15.8446	930.526	0.033
	−39.995	15.8452	930.525	0.033
	−39.995	15.8431	930.523	0.033
	−39.995	15.8436	930.524	0.033
	−30.000	30.5993	929.182	0.034
	−29.999	30.5969	929.179	0.034
	−30.000	30.5980	929.180	0.034
	−30.000	30.5980	929.181	0.034
	−29.999	30.6017	929.182	0.034
	−30.000	25.2379	926.527	0.032
	−30.000	25.2381	926.528	0.032
	−29.999	25.2391	926.527	0.032
	−29.999	25.2392	926.527	0.032
	−30.000	25.2392	926.527	0.032
	−30.000	20.1482	923.945	0.031
	−29.999	20.1496	923.945	0.031
	−29.999	20.1493	923.943	0.031
	−30.000	20.1500	923.944	0.031
	−29.999	20.1501	923.944	0.031
	−29.998	14.9375	921.229	0.030
	−30.000	14.9361	921.230	0.030
	−29.999	14.9371	921.229	0.030
	−29.998	14.9385	921.229	0.030
	−29.999	14.9376	921.230	0.030
	−29.998	10.3699	918.790	0.030
	−30.000	10.3680	918.789	0.030
	−29.999	10.3703	918.791	0.030
	−29.999	10.3715	918.792	0.030
	−29.999	10.3702	918.791	0.030
	−30.000	5.0564	915.876	0.029
	−29.999	5.0586	915.877	0.029
	−30.000	5.0596	915.877	0.029
	−29.999	5.0593	915.877	0.029
	−29.999	5.0596	915.877	0.029
	−29.999	0.8122	913.483	0.029
	−29.999	0.8149	913.485	0.029
	−29.999	0.8131	913.485	0.029
	−29.999	0.8098	913.483	0.029
	−29.999	0.8119	913.483	0.029
	−20.001	14.4426	912.164	0.029
	−19.999	14.4442	912.164	0.029
	−19.999	14.4423	912.162	0.029
	−20.000	14.4418	912.162	0.029
	−19.999	14.4424	912.161	0.029
	−9.999	27.7494	910.898	0.031
	−9.998	27.7489	910.898	0.031
	−9.998	27.7510	910.898	0.031
	−9.998	27.7492	910.898	0.031
	−9.998	27.7478	910.897	0.031
	−10.000	23.7707	908.713	0.030
	−9.997	23.7733	908.713	0.030
	−9.999	23.7708	908.712	0.030
	−9.999	23.7732	908.714	0.030
	−9.997	23.7739	908.713	0.030
	−9.999	18.0750	905.499	0.029
	−9.998	18.0765	905.499	0.029
	−9.997	18.0780	905.499	0.029
	−9.998	18.0770	905.499	0.029
	−9.999	18.0772	905.499	0.029
	−9.998	12.3044	902.132	0.028
	−9.999	12.3005	902.131	0.028
	−9.998	12.2999	902.130	0.028
	−9.998	12.2995	902.130	0.028
	−9.998	12.2991	902.130	0.028
	−9.999	6.2478	898.473	0.027
	−9.999	6.2477	898.472	0.027
	−9.998	6.2502	898.474	0.027
	−9.998	6.2509	898.473	0.027
	−9.999	6.2483	898.472	0.027
	0.012	18.7097	897.243	0.029
	0.012	18.7117	897.245	0.029
	0.013	18.7119	897.243	0.029
	0.012	18.7105	897.243	0.029
	0.012	18.7106	897.244	0.029
	10.000	30.8637	896.062	0.032
	10.002	30.8639	896.060	0.032
	10.002	30.8625	896.060	0.032
	10.002	30.8636	896.061	0.032
	10.002	28.1806	894.476	0.032
	10.003	28.1828	894.476	0.032
	10.003	28.1815	894.474	0.032
	10.002	28.1810	894.475	0.032
	10.003	28.1819	894.475	0.032
	10.002	25.8708	893.090	0.031
	10.003	25.8709	893.090	0.031
	10.003	25.8691	893.088	0.031
	10.002	25.8695	893.089	0.031
	10.002	25.8705	893.090	0.031
	10.002	23.8631	891.870	0.030
	10.002	23.8594	891.869	0.030
	10.002	23.8583	891.869	0.030
	10.003	23.8609	891.869	0.030
	10.002	23.8598	891.869	0.030
	10.001	21.0399	890.130	0.030
	10.001	21.0401	890.130	0.030
	10.002	21.0411	890.131	0.030
	10.002	21.0419	890.131	0.030
	10.002	21.0417	890.129	0.030
	10.001	17.9894	888.213	0.029
	10.002	17.9863	888.210	0.029
	10.001	17.9866	888.212	0.029
	10.002	17.9878	888.212	0.029
	10.003	17.9908	888.213	0.029
	10.002	15.9128	886.886	0.029
	10.003	15.9118	886.885	0.029
	10.003	15.9159	886.886	0.029
	10.002	15.9183	886.889	0.029
	10.003	15.9191	886.889	0.029
	10.002	12.4496	884.632	0.028
	10.002	12.4507	884.632	0.028
	10.002	12.4512	884.632	0.028
	10.003	12.4535	884.633	0.028
	10.003	12.4526	884.633	0.028
	10.002	9.9215	882.952	0.028
	10.003	9.9227	882.952	0.028
	10.003	9.9226	882.952	0.028
	10.003	9.9227	882.953	0.028
	10.003	9.9228	882.953	0.028
	10.001	8.0081	881.661	0.028
	10.002	8.0097	881.661	0.028
	10.003	8.0096	881.661	0.028
	10.003	8.0111	881.662	0.028
	10.002	8.0108	881.662	0.028
	10.002	5.0658	879.638	0.027
	10.003	5.0663	879.638	0.027
	10.003	5.0670	879.638	0.027
	10.002	5.0640	879.637	0.027
	10.002	5.0615	879.636	0.027
	10.002	3.0677	878.240	0.027
	10.003	3.0716	878.242	0.027
	10.002	3.0696	878.241	0.027
	10.002	3.0697	878.241	0.027
	10.003	3.0720	878.242	0.027
	10.002	0.5198	876.425	0.027
	10.003	0.5205	876.425	0.027
	10.002	0.5194	876.425	0.027
	10.002	0.5201	876.425	0.027
	10.003	0.5194	876.424	0.027
	20.002	11.5906	875.254	0.029
	20.002	11.5908	875.254	0.029
	20.002	11.5921	875.255	0.029
	20.001	11.5908	875.254	0.029
	20.002	11.5922	875.254	0.029
	30.001	22.4315	874.124	0.032
	30.002	22.4302	874.122	0.032
	30.002	22.4314	874.122	0.032
	30.001	22.4291	874.122	0.032
	30.001	22.4286	874.121	0.032
	40.001	33.0667	873.039	0.035
	40.001	33.0669	873.039	0.035
	40.001	33.0680	873.039	0.035
	40.001	33.0661	873.039	0.035
	40.001	33.0639	873.037	0.035
	40.002	29.2719	870.486	0.034
	40.001	29.2706	870.486	0.034
	40.002	29.2715	870.485	0.034
	40.002	29.2721	870.485	0.034
	40.002	29.2728	870.486	0.034
	40.002	27.0272	868.945	0.034
	40.002	27.0282	868.944	0.034
	40.001	27.0277	868.944	0.034
	40.003	27.0292	868.945	0.034
	40.003	27.0287	868.945	0.034
	40.001	23.9812	866.813	0.033
	40.002	23.9828	866.813	0.033
	40.003	23.9830	866.812	0.033
	40.002	23.9813	866.812	0.033
	40.001	23.9818	866.812	0.033
	40.001	21.1388	864.779	0.032
	40.000	21.1380	864.780	0.032
	40.000	21.1378	864.779	0.032
	40.002	21.1394	864.779	0.032
	40.000	21.1376	864.779	0.032
	40.001	18.0243	862.499	0.032
	40.001	18.0243	862.500	0.032
	40.002	18.0239	862.499	0.032
	40.002	18.0247	862.499	0.032
	40.001	18.0243	862.498	0.032
	40.001	15.0817	860.294	0.031
	40.001	15.0818	860.294	0.031
	40.001	15.0829	860.295	0.031
	40.002	15.0837	860.296	0.031
	40.001	15.0827	860.295	0.031
	40.002	12.0138	857.938	0.031
	40.001	12.0134	857.938	0.031
	40.001	12.0125	857.936	0.031
	40.002	12.0133	857.935	0.031
	40.002	12.0127	857.936	0.031
	40.001	9.0991	855.639	0.030
	40.001	9.0980	855.639	0.030
	40.002	9.0973	855.639	0.030
	40.002	9.0978	855.637	0.030
	40.001	9.0973	855.637	0.030
	40.000	6.0393	853.163	0.030
	40.001	6.0394	853.163	0.030
	40.002	6.0397	853.162	0.030
	40.002	6.0396	853.162	0.030
	40.001	6.0388	853.161	0.030
	40.000	2.9800	850.616	0.030
	40.001	2.9801	850.615	0.030
	40.002	2.9809	850.615	0.030
	40.002	2.9812	850.616	0.030
	40.001	2.9801	850.616	0.030
	40.001	0.6598	848.634	0.030
	40.001	0.6592	848.634	0.030
	40.002	0.6596	848.633	0.030
	40.001	0.6598	848.634	0.030
	40.000	0.6615	848.635	0.030
	49.998	10.1894	847.590	0.032
	50.001	10.1912	847.589	0.032
	50.001	10.1922	847.589	0.032
	50.001	10.1922	847.588	0.032
	50.001	10.1925	847.590	0.032
	60.001	19.5563	846.580	0.034
	60.001	19.5563	846.579	0.034
	60.002	19.5572	846.579	0.034
	60.002	19.5568	846.579	0.034
	60.001	19.5572	846.579	0.034
	60.001	15.2272	842.992	0.034
	60.002	15.2319	842.993	0.034
	60.002	15.2319	842.994	0.034
	60.002	15.2320	842.994	0.034
	60.003	15.2330	842.994	0.034
	60.002	10.0412	838.495	0.033
	60.002	10.0410	838.495	0.033
	60.002	10.0411	838.494	0.033
	60.003	10.0424	838.496	0.033
	60.003	10.0418	838.495	0.033
	60.002	5.1271	834.013	0.032
	60.002	5.1264	834.011	0.032
	60.003	5.1284	834.013	0.032
	60.003	5.1289	834.015	0.032
	60.003	5.1282	834.013	0.032
	60.002	0.6483	829.712	0.032
	60.001	0.6480	829.714	0.032
	60.002	0.6480	829.713	0.032
	60.003	0.6491	829.713	0.032
	60.002	0.6481	829.714	0.032
	70.002	9.2470	828.727	0.034
	70.001	9.2479	828.728	0.034
	70.003	9.2488	828.728	0.034
	70.003	9.2480	828.727	0.034
	70.002	9.2477	828.727	0.034
	80.002	17.7099	827.773	0.036
	80.003	17.7103	827.773	0.036
	80.004	17.7111	827.773	0.036
	80.004	17.7110	827.773	0.036
	80.003	17.7098	827.773	0.036
	80.002	14.0712	824.354	0.036
	80.004	14.0738	824.356	0.036
	80.003	14.0734	824.355	0.036
	80.003	14.0736	824.356	0.036
	80.004	14.0743	824.356	0.036
	80.004	10.0714	820.434	0.035
	80.003	10.0715	820.434	0.035
	80.004	10.0723	820.434	0.035
	80.004	10.0729	820.435	0.035
	80.003	10.0723	820.435	0.035
	80.003	5.0880	815.286	0.035
	80.003	5.0890	815.287	0.035
	80.004	5.0891	815.285	0.035
	80.004	5.0881	815.285	0.035
	80.003	5.0885	815.285	0.035
	80.003	0.5582	810.313	0.035
	80.004	0.5582	810.312	0.035
	80.004	0.5583	810.310	0.035
	80.003	0.5576	810.311	0.035
	80.003	0.5567	810.311	0.035
	90.002	8.3064	809.389	0.037
	90.003	8.3062	809.387	0.037
	90.003	8.3063	809.387	0.037
	90.004	8.3080	809.388	0.037
	90.005	8.3073	809.387	0.037
	100.003	15.9451	808.490	0.039
	100.005	15.9460	808.491	0.039
	100.006	15.9464	808.491	0.039
	100.005	15.9461	808.491	0.039
	100.004	15.9455	808.491	0.039
	100.005	12.1105	804.352	0.038
	100.004	12.1095	804.351	0.038
	100.005	12.1099	804.351	0.038
	100.006	12.1113	804.352	0.038
	100.005	12.1119	804.353	0.038
	100.005	8.2213	799.937	0.038
	100.005	8.2214	799.937	0.038
	100.006	8.2218	799.935	0.038
	100.005	8.2212	799.937	0.038
	100.005	8.2207	799.937	0.038
	100.005	4.1477	795.045	0.037
	100.005	4.1469	795.046	0.037
	100.004	4.1463	795.044	0.037
	100.005	4.1467	795.044	0.037
	100.006	4.1470	795.044	0.037
	100.004	0.5183	790.422	0.037
	100.004	0.5173	790.421	0.037
	100.005	0.5180	790.422	0.037
	100.006	0.5180	790.421	0.037
	100.004	0.5171	790.419	0.037
	110.005	7.4964	789.570	0.039
	110.005	7.4973	789.569	0.039
	110.007	7.4982	789.571	0.039
	110.006	7.4977	789.569	0.039
	110.005	7.4977	789.570	0.039
	115.006	10.9518	789.150	0.040
	115.007	10.9532	789.151	0.040
	115.007	10.9528	789.150	0.040
	115.007	10.9529	789.151	0.040
	115.007	10.9529	789.150	0.040
	120.006	14.3813	788.734	0.041
	120.007	14.3829	788.733	0.041
	120.004	14.3791	788.733	0.041
	120.003	14.3780	788.732	0.041
	120.003	14.3788	788.731	0.041
	120.004	14.3805	788.733	0.041
	120.004	14.3842	788.739	0.041
	125.003	17.7973	788.335	0.042
	125.005	17.7999	788.335	0.042
	125.006	17.8008	788.335	0.042
	125.005	17.8007	788.336	0.042
	125.004	17.7998	788.334	0.042
	130.004	21.1756	787.916	0.043
	130.004	21.1749	787.916	0.043
	130.005	21.1758	787.916	0.043
	130.005	21.1754	787.916	0.043
	130.004	21.1741	787.914	0.043
	135.005	24.5319	787.505	0.044
	135.005	24.5318	787.506	0.044
	135.006	24.5321	787.504	0.044
	135.005	24.5320	787.505	0.044
	135.005	24.5318	787.504	0.044
	140.005	27.8683	787.101	0.045
	140.007	27.8689	787.099	0.045
	140.006	27.8690	787.100	0.045
	140.005	27.8697	787.103	0.045
	140.006	27.8704	787.102	0.045
	145.005	31.1942	786.714	0.046
	145.006	31.1930	786.712	0.046
	145.007	31.1935	786.711	0.046
	145.008	31.1929	786.709	0.046
	145.007	31.1918	786.709	0.046
	150.007	34.3186	786.130	0.047
	150.008	34.2917	786.100	0.047
	150.007	34.2797	786.087	0.047
	150.007	34.2691	786.075	0.047
	150.008	34.2584	786.061	0.047
	150.008	29.9475	781.187	0.046
	150.007	29.9386	781.178	0.046
	150.006	29.9322	781.171	0.046
	150.008	29.9264	781.162	0.046
	150.008	29.9205	781.156	0.046
	150.009	26.9681	777.657	0.046
	150.008	26.9621	777.650	0.046
	150.007	26.9584	777.646	0.046
	150.008	26.9558	777.642	0.046
	150.009	26.9529	777.639	0.046
	150.008	24.0254	774.027	0.045
	150.008	24.0142	774.012	0.045
	150.008	24.0127	774.010	0.045
	150.009	24.0108	774.008	0.045
	150.009	24.0078	774.004	0.045
	150.009	20.9296	770.037	0.045
	150.008	20.9281	770.035	0.045
	150.008	20.9264	770.034	0.045
	150.009	20.9252	770.031	0.045
	150.009	20.9242	770.029	0.045
	150.008	18.0045	766.091	0.044
	150.008	18.0022	766.086	0.044
	150.008	18.0014	766.086	0.044
	150.009	18.0015	766.085	0.044
	150.010	18.0011	766.084	0.044
	150.009	14.9905	761.818	0.044
	150.008	14.9888	761.817	0.044
	150.009	14.9884	761.815	0.044
	150.009	14.9884	761.813	0.044
	150.009	14.9880	761.814	0.044
	150.008	12.0432	757.415	0.043
	150.008	12.0430	757.414	0.043
	150.008	12.0433	757.413	0.043
	150.008	12.0423	757.412	0.043
	150.008	12.0423	757.413	0.043
	150.008	8.9974	752.595	0.043
	150.008	8.9974	752.595	0.043
	150.009	8.9971	752.594	0.043
	150.009	8.9969	752.593	0.043
	150.008	8.9957	752.593	0.043
	150.007	6.0425	747.616	0.042
	150.008	6.0433	747.618	0.042
	150.009	6.0438	747.618	0.042
	150.008	6.0429	747.616	0.042
	150.008	6.0427	747.617	0.042
	150.007	3.0537	742.226	0.042
	150.007	3.0535	742.226	0.042
	150.008	3.0542	742.225	0.042
	150.008	3.0538	742.225	0.042
	150.007	3.0540	742.225	0.042
	150.009	0.5414	737.362	0.042
	150.008	0.5407	737.362	0.042
	150.008	0.5411	737.362	0.042
	150.009	0.5413	737.362	0.042
	150.008	0.5406	737.362	0.042
Filling 2a				
	40.000	7.7237	854.535	0.030
	40.002	7.7249	854.535	0.030
	40.001	7.7245	854.535	0.030
	40.001	7.7242	854.535	0.030
	40.002	7.7251	854.535	0.030
Filling 3				
	29.997	29.9617	879.126	0.033
	29.999	29.9633	879.125	0.033
	29.999	29.9607	879.125	0.033
	30.000	29.9601	879.123	0.033
	30.001	29.9588	879.122	0.033
	29.999	25.0914	875.922	0.032
	30.000	25.0912	875.921	0.032
	30.001	25.0919	875.921	0.032
	30.000	25.0903	875.920	0.032
	30.000	25.0906	875.919	0.032
	30.000	20.1581	872.566	0.031
	30.001	20.1592	872.565	0.031
	30.001	20.1595	872.566	0.031
	30.001	20.1591	872.565	0.031
	30.001	20.1574	872.565	0.031
	30.000	15.2638	869.116	0.030
	30.001	15.2657	869.117	0.030
	30.002	15.2650	869.116	0.030
	30.002	15.2648	869.116	0.030
	30.001	15.2643	869.115	0.030
	30.000	10.1390	865.363	0.029
	30.000	10.1390	865.363	0.029
	30.001	10.1406	865.364	0.029
	30.001	10.1399	865.363	0.029
	30.001	10.1394	865.361	0.029
	30.000	5.0194	861.451	0.029
	30.000	5.0200	861.451	0.029
	30.001	5.0214	861.452	0.029
	30.001	5.0218	861.452	0.029
	30.001	5.0209	861.452	0.029
	30.001	0.7644	858.066	0.029
	30.001	0.7648	858.066	0.029
	30.000	0.7635	858.066	0.029
	30.001	0.7644	858.066	0.029
	30.002	0.7643	858.066	0.029
	40.000	10.7884	856.979	0.031
	40.000	10.7922	856.983	0.031
	40.000	10.7919	856.982	0.031
	40.000	10.7946	856.985	0.031
	40.000	10.7974	856.985	0.031
	50.000	20.6246	855.930	0.033
	50.000	20.6247	855.930	0.033
	50.000	20.6227	855.928	0.033
	50.001	20.6240	855.929	0.033
	50.001	20.6227	855.927	0.033
	59.999	30.2738	854.908	0.037
	59.999	30.2788	854.912	0.037
	60.000	30.2822	854.913	0.037
	60.001	30.2817	854.913	0.037
	60.000	30.2843	854.915	0.037
	59.999	27.0542	852.483	0.036
	60.000	27.0563	852.483	0.036
	60.001	27.0538	852.481	0.036
	59.999	27.0523	852.481	0.036
	60.001	27.0550	852.482	0.036
	59.999	23.7636	849.938	0.035
	60.001	23.7635	849.936	0.035
	60.002	23.7573	849.931	0.035
	60.001	23.7550	849.929	0.035
	60.001	23.7544	849.929	0.035
	60.000	19.7665	846.751	0.034
	60.000	19.7624	846.746	0.034
	60.000	19.7619	846.745	0.034
	60.001	19.7620	846.745	0.034
	60.002	19.7623	846.745	0.034
	60.001	15.1296	842.908	0.033
	60.001	15.1271	842.907	0.033
	60.001	15.1268	842.905	0.033
	60.001	15.1268	842.906	0.033
	60.001	15.1272	842.906	0.033
	69.999	24.1977	841.929	0.036
	70.001	24.1982	841.927	0.036
	70.001	24.1988	841.927	0.036
	70.002	24.1988	841.925	0.036
	70.002	24.2000	841.928	0.036
	80.001	33.0852	840.951	0.040
	80.001	33.0854	840.951	0.040
	80.003	33.0863	840.951	0.040
	80.003	33.0852	840.949	0.040
	80.002	33.0840	840.948	0.040
	80.002	29.8274	838.309	0.039
	80.003	29.8279	838.309	0.039
	80.004	29.8292	838.310	0.039
	80.003	29.8288	838.310	0.039
	80.003	29.8294	838.310	0.039
	80.003	25.1394	834.373	0.038
	80.003	25.1364	834.371	0.038
	80.004	25.1369	834.371	0.038
	80.004	25.1369	834.372	0.038
	80.004	25.1368	834.371	0.038
	80.004	19.9546	829.818	0.037
	80.004	19.9530	829.816	0.037
	80.005	19.9495	829.812	0.037
	80.004	19.9472	829.810	0.037
	80.004	19.9452	829.809	0.037
	80.003	15.0964	825.330	0.036
	80.002	15.0934	825.327	0.036
	80.003	15.0935	825.327	0.036
	80.004	15.0938	825.326	0.036
	80.003	15.0918	825.326	0.036
	90.002	23.3250	824.387	0.039
	90.002	23.3252	824.388	0.039
	90.004	23.3257	824.387	0.039
	90.004	23.3237	824.385	0.039
	90.003	23.3223	824.384	0.039
	100.003	31.4102	823.456	0.042
	100.003	31.4092	823.454	0.042
	100.004	31.4095	823.454	0.042
	100.005	31.4079	823.453	0.042
	100.004	31.4066	823.453	0.042
	100.004	28.3371	820.671	0.041
	100.003	28.3347	820.670	0.041
	100.004	28.3351	820.669	0.041
	100.005	28.3342	820.669	0.041
	100.003	28.3321	820.667	0.041
	100.003	24.1976	816.784	0.040
	100.003	24.1969	816.783	0.040
	100.004	24.1981	816.784	0.040
	100.004	24.1977	816.784	0.040
	100.003	24.1966	816.783	0.040
	100.003	20.5655	813.229	0.040
	100.003	20.5650	813.229	0.040
	100.004	20.5655	813.228	0.040
	100.004	20.5659	813.230	0.040
	100.003	20.5639	813.228	0.040
	100.003	15.1512	807.651	0.039
	100.003	15.1515	807.651	0.039
	100.004	15.1532	807.653	0.039
	100.003	15.1536	807.652	0.039
	100.003	15.1533	807.654	0.039
	110.000	22.6514	806.778	0.041
	110.001	22.6517	806.779	0.041
	110.002	22.6521	806.779	0.041
	110.001	22.6514	806.779	0.041
	110.001	22.6502	806.777	0.041
	120.000	30.0265	805.908	0.044
	120.001	30.0241	805.905	0.044
	120.002	30.0239	805.903	0.044
	120.003	30.0227	805.901	0.044
	120.003	30.0218	805.900	0.044
	120.002	25.0898	800.840	0.043
	120.002	25.0879	800.837	0.043
	120.002	25.0870	800.836	0.043
	120.003	25.0880	800.837	0.043
	120.003	25.0868	800.836	0.043
	120.001	20.2062	795.528	0.042
	120.003	20.2079	795.528	0.042
	120.002	20.2052	795.527	0.042
	120.001	20.2028	795.525	0.042
	120.001	20.2025	795.525	0.042
	120.002	14.9413	789.414	0.041
	120.001	14.9389	789.413	0.041
	120.002	14.9386	789.411	0.041
	120.002	14.9397	789.413	0.041
	120.002	14.9377	789.411	0.041
	120.001	2.5228	772.912	0.040
	120.001	2.5230	772.914	0.040
	120.002	2.5244	772.914	0.040
	120.003	2.5243	772.913	0.040
	120.002	2.5246	772.911	0.040
Filling 3a				
	39.999	6.8149	853.798	0.030
	39.999	6.8149	853.798	0.030
	39.999	6.8134	853.796	0.030
	40.000	6.8154	853.797	0.030
	40.000	6.8135	853.795	0.030
	79.999	30.0548	838.496	0.039
	80.000	30.0556	838.496	0.039
	80.000	30.0545	838.495	0.039
	80.001	30.0545	838.495	0.039
	80.001	30.0531	838.494	0.039
	79.997	0.8710	810.672	0.035
	80.001	0.8754	810.673	0.035
	80.002	0.8770	810.673	0.035
	80.000	0.8760	810.673	0.035
	80.000	0.8753	810.673	0.035
	100.002	16.2965	808.862	0.039
	100.002	16.2960	808.861	0.039
	100.003	16.2962	808.859	0.039
	100.003	16.2964	808.860	0.039
	100.002	16.2962	808.860	0.039
	120.002	31.2424	807.111	0.044
	120.001	31.2414	807.111	0.044
	120.001	31.2406	807.110	0.044
	120.002	31.2399	807.108	0.044
	120.002	31.2348	807.103	0.044
	120.002	10.0450	783.303	0.040
	120.002	10.0437	783.302	0.040
	120.001	10.0426	783.299	0.040
	120.002	10.0437	783.300	0.040
	120.003	10.0443	783.300	0.040
	120.001	5.0864	776.608	0.040
	120.002	5.0858	776.607	0.040
	120.001	5.0851	776.608	0.040
	120.002	5.0858	776.605	0.040
	120.002	5.0858	776.606	0.040
	119.999	0.7068	770.181	0.039
	120.000	0.7057	770.179	0.039
	120.001	0.7057	770.177	0.039
	120.001	0.7051	770.178	0.039
	120.001	0.7053	770.177	0.039
	125.001	3.8524	769.788	0.040
	125.002	3.8547	769.791	0.040
	125.002	3.8559	769.792	0.040
	125.002	3.8564	769.794	0.040
	125.003	3.8550	769.790	0.040
	130.003	6.9721	769.386	0.041
	130.003	6.9721	769.387	0.041
	130.003	6.9722	769.385	0.041
	130.004	6.9726	769.387	0.041
	130.004	6.9720	769.385	0.041
	135.004	10.0740	768.990	0.042
	135.003	10.0734	768.990	0.042
	135.003	10.0739	768.990	0.042
	135.005	10.0745	768.990	0.042
	135.004	10.0739	768.989	0.042
	140.004	13.1552	768.594	0.043
	140.004	13.1554	768.595	0.043
	140.003	13.1535	768.593	0.043
	140.004	13.1543	768.592	0.043
	140.004	13.1537	768.591	0.043
	140.003	13.1538	768.593	0.043
	145.004	16.2160	768.199	0.043
	145.004	16.2146	768.197	0.043
	145.005	16.2145	768.197	0.043
	145.004	16.2137	768.196	0.043
	145.004	16.2138	768.197	0.043
	150.002	19.2622	767.814	0.044
	150.002	19.2637	767.816	0.044
	150.002	19.2650	767.818	0.044
	150.003	19.2653	767.817	0.044
	150.003	19.2638	767.815	0.044
	160.003	25.2903	767.037	0.046
	160.005	25.2901	767.036	0.046
	160.004	25.2890	767.034	0.046
	160.004	25.2877	767.033	0.046
	160.004	25.2872	767.031	0.046
	165.004	28.2650	766.638	0.047
	165.004	28.2652	766.638	0.047
	165.004	28.2622	766.634	0.047
	165.004	28.2620	766.634	0.047
	165.005	28.2622	766.634	0.047
	175.007	33.9837	765.629	0.050
	175.007	33.9536	765.593	0.050
	175.008	33.9411	765.576	0.050
	175.009	33.9269	765.557	0.050
	175.008	33.9108	765.539	0.050
	175.006	25.9731	755.042	0.048
	175.007	25.9588	755.021	0.048
	175.006	25.9541	755.015	0.048
	175.007	25.9512	755.010	0.048
	175.008	25.9468	755.003	0.048
	175.006	22.1998	749.576	0.047
	175.006	22.1933	749.566	0.047
	175.006	22.1906	749.560	0.047
	175.007	22.1891	749.559	0.047
	175.008	22.1874	749.555	0.047
	175.007	22.1851	749.554	0.047
	175.006	17.9461	742.960	0.046
	175.007	17.9458	742.960	0.046
	175.006	17.9426	742.955	0.046
	175.007	17.9423	742.954	0.046
	175.007	17.9407	742.953	0.046
	185.006	23.2286	742.215	0.048
	185.007	23.2268	742.211	0.048
	185.008	23.2264	742.209	0.048
	185.007	23.2241	742.206	0.048
	185.007	23.2224	742.204	0.048
	190.009	25.8354	741.821	0.050
	190.008	25.8323	741.818	0.050
	190.009	25.8311	741.815	0.050
	190.009	25.8300	741.814	0.050
	190.009	25.8284	741.811	0.050
	200.010	31.0194	741.069	0.053
	200.010	31.0174	741.067	0.053
	200.011	31.0162	741.064	0.053
	200.012	31.0136	741.059	0.053
	200.010	19.7931	722.679	0.051
	200.013	19.7929	722.676	0.051
	200.012	19.7919	722.674	0.051
	200.011	19.7917	722.675	0.051
	200.013	19.7916	722.673	0.051
	200.011	10.0529	702.521	0.049
	200.012	10.0522	702.520	0.049
	200.011	10.0515	702.520	0.049
	200.012	10.0519	702.518	0.049
	200.012	1.2074	677.755	0.047
	200.012	1.2072	677.755	0.047
	200.012	1.2077	677.756	0.047
	200.013	1.2076	677.754	0.047
	200.013	1.2074	677.754	0.047
Filling 3 b				
	40.000	2.1452	849.906	0.030
	40.000	2.1447	849.906	0.030
	39.999	2.1442	849.906	0.030
	40.000	2.1459	849.906	0.030
	40.000	2.1452	849.905	0.030

**Table A2:** Experimentally measured temperatures *t*, pressures *p*, and densities *ρ*_exp_ for degassed toluene with the standard uncertainty *u* (*ρ*_fluid_); measurements carried out in 2007

	*t* (°C)	*p* (MPa)	*ρ*_exp_ (kg m^−3^)	*u* (*ρ*_fluid_) (kg m^−3^)
Filling 1				
	−39.997	15.5527	930.436	0.033
	−39.998	15.5480	930.434	0.033
	−39.998	15.5451	930.432	0.033
	−39.998	15.5409	930.430	0.033
	−40.000	7.8831	926.526	0.032
	−39.999	7.8898	926.529	0.032
	−39.998	7.8887	926.528	0.032
	−39.998	7.8846	926.525	0.032
	−39.998	4.2666	924.626	0.032
	−39.999	4.2674	924.628	0.032
	−39.998	4.2682	924.627	0.032
	−39.999	4.2670	924.627	0.032
	−40.000	0.8126	922.781	0.031
	−39.998	0.8148	922.783	0.031
	−39.998	0.8171	922.785	0.031
	−39.999	0.8159	922.784	0.031
	−20.009	29.0323	920.043	0.031
	−20.008	29.0385	920.044	0.031
	−20.007	29.0396	920.045	0.031
	−20.007	29.0390	920.042	0.031
	−20.007	26.4114	918.680	0.031
	−20.006	26.4135	918.680	0.031
	−20.005	26.4125	918.681	0.031
	−20.007	26.4091	918.679	0.031
	−20.007	19.5375	915.026	0.029
	−20.006	19.5397	915.026	0.029
	−20.006	19.5404	915.028	0.029
	−20.005	19.5418	915.026	0.029
	−20.004	11.9133	910.812	0.028
	−20.006	11.9124	910.811	0.028
	−20.005	11.9102	910.809	0.028
	−20.004	11.9097	910.807	0.028
	−20.006	3.6629	906.040	0.027
	−20.005	3.6685	906.042	0.027
	−20.005	3.6685	906.042	0.027
	−20.006	3.6651	906.041	0.027
	−20.006	0.5244	904.163	0.027
	−20.006	0.5208	904.158	0.027
	−20.005	0.5281	904.164	0.027
	−20.005	0.5259	904.161	0.027
	−0.005	25.9724	901.585	0.030
	−0.003	25.9741	901.585	0.030
	−0.003	25.9724	901.583	0.030
	−0.003	25.9730	901.584	0.030
	−0.003	20.5865	898.437	0.028
	−0.003	20.5868	898.437	0.028
	−0.001	20.5882	898.436	0.028
	−0.002	20.5839	898.434	0.028
	−0.002	15.7960	895.548	0.028
	−0.003	15.7957	895.549	0.028
	−0.002	15.7962	895.551	0.028
	−0.002	15.7938	895.547	0.028
	−0.003	7.8170	890.543	0.027
	−0.002	7.8203	890.544	0.027
	−0.002	7.8215	890.546	0.027
	−0.001	7.8232	890.547	0.027
	19.999	31.2309	888.146	0.032
	20.000	31.2284	888.145	0.032
	20.000	31.2275	888.144	0.032
	20.000	31.2257	888.142	0.032
	19.999	27.5973	885.895	0.031
	19.999	27.5933	885.892	0.031
	20.000	27.5929	885.892	0.031
	20.001	27.5917	885.890	0.031
	20.000	20.3644	881.254	0.029
	20.001	20.3635	881.254	0.029
	20.000	20.3615	881.253	0.029
	20.000	20.3629	881.253	0.029
	20.000	11.6260	875.333	0.028
	20.000	11.6257	875.333	0.028
	20.001	11.6313	875.336	0.028
	20.000	11.6279	875.334	0.028
	19.999	4.0074	869.849	0.027
	19.999	4.0093	869.850	0.027
	20.001	4.0098	869.850	0.027
	20.001	4.0100	869.849	0.027
	20.000	1.2611	867.787	0.027
	20.000	1.2582	867.784	0.027
	20.000	1.2570	867.782	0.027
	20.000	1.2572	867.780	0.027
	49.996	32.1864	864.433	0.035
	49.996	32.1857	864.432	0.035
	49.997	32.1889	864.434	0.035
	49.998	32.1914	864.434	0.035
	50.001	27.9027	861.384	0.034
	50.000	27.9014	861.384	0.034
	49.999	27.9005	861.383	0.034
	50.000	27.9014	861.384	0.034
	49.999	19.5104	855.126	0.032
	50.001	19.5104	855.121	0.032
	50.001	19.5122	855.125	0.032
	50.000	19.5118	855.125	0.032
	50.001	11.9712	849.117	0.031
	50.000	11.9695	849.116	0.031
	50.000	11.9692	849.115	0.031
	50.001	11.9696	849.115	0.031
	49.999	4.2940	842.557	0.030
	50.001	4.2950	842.557	0.030
	50.001	4.2943	842.557	0.030
	50.001	4.2940	842.557	0.030
	49.999	1.3048	839.865	0.030
	50.001	1.3056	839.865	0.030
	50.000	1.3044	839.863	0.030
	50.000	1.3044	839.862	0.030
	79.996	27.9725	836.822	0.038
	79.997	27.9729	836.823	0.038
	79.998	27.9729	836.821	0.038
	79.998	27.9757	836.824	0.038
	79.999	27.9757	836.823	0.038
	79.999	27.9750	836.821	0.038
	80.000	27.9765	836.822	0.038
	80.000	27.9761	836.822	0.038
	80.000	19.6924	829.628	0.036
	79.999	19.6907	829.627	0.036
	79.999	19.6916	829.628	0.036
	80.000	19.6937	829.629	0.036
	79.998	11.5709	821.971	0.035
	80.000	11.5716	821.970	0.035
	79.999	11.5711	821.971	0.035
	79.999	11.5711	821.970	0.035
	79.997	3.7114	813.854	0.034
	79.999	3.7131	813.853	0.034
	79.999	3.7100	813.851	0.034
	79.999	3.7099	813.850	0.034
	79.999	1.2011	811.081	0.034
	79.998	1.1996	811.082	0.034
	79.999	1.2002	811.081	0.034
	80.000	1.1997	811.081	0.034
	99.993	16.6017	809.228	0.038
	99.995	16.6061	809.230	0.038
	99.997	16.6061	809.229	0.038
	99.996	16.6058	809.230	0.038
	119.997	31.5447	807.449	0.043
	119.997	31.5429	807.448	0.043
	119.998	31.5447	807.449	0.043
	119.998	31.5448	807.448	0.043
	119.996	27.7615	803.659	0.042
	119.998	27.7598	803.653	0.042
	119.997	27.7580	803.652	0.042
	119.998	27.7580	803.651	0.042
	119.997	20.2707	795.639	0.041
	119.998	20.2717	795.639	0.041
	119.998	20.2706	795.639	0.041
	119.998	20.2705	795.638	0.041
	119.997	11.7558	785.529	0.039
	119.997	11.7565	785.531	0.039
	119.997	11.7582	785.530	0.039
	119.998	11.7595	785.533	0.039
	119.998	4.0985	775.241	0.039
	119.998	4.0985	775.243	0.039
	119.997	4.0981	775.242	0.039
	119.998	4.0944	775.236	0.039
	119.997	0.7124	770.226	0.038
	119.997	0.7132	770.229	0.038
	119.997	0.7131	770.226	0.038
	119.997	0.7142	770.228	0.038
	149.997	19.2419	767.823	0.043
	149.997	19.2422	767.823	0.043
	149.997	19.2423	767.824	0.043
	149.997	19.2421	767.823	0.043
	149.997	19.2423	767.823	0.043
	149.998	19.2436	767.824	0.043
	149.997	19.2435	767.826	0.043
	149.998	19.2438	767.825	0.043
	149.999	11.6928	756.915	0.042
	149.998	11.6928	756.915	0.042
	149.998	11.6916	756.913	0.042
	149.998	11.6925	756.915	0.042
	149.998	4.0855	744.172	0.041
	149.998	4.0847	744.171	0.041
	149.998	4.0836	744.166	0.041
	149.998	4.0833	744.168	0.041
	149.999	1.0294	738.375	0.041
	149.998	1.0290	738.376	0.041
	149.998	1.0274	738.370	0.041
	149.998	1.0265	738.368	0.041
Filling 2				
	−39.997	13.9488	929.635	0.033
	−39.996	13.9478	929.634	0.033
	−39.996	13.9488	929.634	0.033
	−39.997	13.9484	929.637	0.033
	−39.997	6.3753	925.740	0.032
	−39.997	6.3740	925.742	0.032
	−39.997	6.3777	925.743	0.032
	−39.997	6.3782	925.743	0.032
	−39.998	1.1573	922.970	0.031
	−39.997	1.1615	922.973	0.031
	−39.998	1.1611	922.974	0.031
	−39.998	1.1655	922.974	0.031
	−20.010	29.3904	920.233	0.032
	−20.008	29.3994	920.235	0.032
	−20.007	29.4014	920.236	0.032
	−20.007	29.4054	920.237	0.032
	−20.008	11.9975	910.865	0.028
	−20.005	11.9997	910.863	0.028
	−20.005	11.9982	910.864	0.028
	−20.005	12.0003	910.864	0.028
	79.991	12.0733	822.481	0.035
	79.997	12.0692	822.469	0.035
	79.998	12.0665	822.465	0.035
	79.998	12.0637	822.464	0.035
	79.998	12.0523	822.452	0.035
	79.999	12.0521	822.450	0.035
	79.999	12.0486	822.448	0.035
	79.999	12.0459	822.445	0.035
	79.997	7.9338	818.319	0.035
	79.999	7.9354	818.319	0.035
	79.999	7.9349	818.320	0.035
	79.998	7.9341	818.318	0.035
	79.998	4.0220	814.197	0.034
	79.999	4.0232	814.197	0.034
	79.998	4.0190	814.197	0.034
	79.998	4.0175	814.190	0.034
	79.998	1.0563	810.930	0.034
	79.997	1.0556	810.927	0.034
	79.999	1.0567	810.927	0.034
	79.999	1.0546	810.925	0.034

**Table A3:** Experimentally measured temperatures *t*, pressures *p*, and densities *ρ*_exp_ for the air saturated toluene with the the standard uncertainty *u* (*ρ*_fluid_); measurements carried out in 2007

	*t* (°C)	*p* (MPa)	*ρ*_exp_ (kg m^−3^)	*u* (*ρ*_fluid_) (kg m^−3^)
Filling 1				
	−39.999	18.8499	932.043	0.033
	−39.999	18.8446	932.041	0.033
	−39.999	18.8418	932.040	0.033
	−39.999	18.8402	932.037	0.033
	−40.000	6.4334	925.734	0.032
	−39.999	6.4368	925.734	0.032
	−39.999	6.4361	925.733	0.032
	−39.999	6.4336	925.731	0.032
	−40.001	0.8799	922.777	0.031
	−39.999	0.8807	922.777	0.031
	−40.000	0.8839	922.780	0.031
	−40.000	0.8852	922.779	0.031
	−20.003	29.0999	920.032	0.031
	−20.003	29.0956	920.034	0.031
	−20.002	29.1042	920.035	0.031
	−20.002	29.1055	920.036	0.031
	−20.002	21.5364	916.057	0.030
	−20.002	21.5273	916.050	0.030
	−20.001	21.5342	916.055	0.030
	−20.001	21.5294	916.050	0.030
	−20.001	15.6096	912.828	0.029
	−20.001	15.6120	912.830	0.029
	−20.002	15.6091	912.828	0.029
	−20.001	15.6124	912.830	0.029
	−20.002	7.7837	908.401	0.028
	−20.002	7.7850	908.402	0.028
	−20.001	7.7883	908.402	0.028
	−20.001	7.7888	908.401	0.028
	−20.003	1.8406	904.900	0.027
	−20.001	1.8377	904.898	0.027
	−20.001	1.8457	904.902	0.027
	−20.002	1.8459	904.903	0.027
	−0.005	27.3479	902.327	0.030
	−0.004	27.3536	902.328	0.030
	−0.004	27.3524	902.328	0.030
	−0.004	27.3522	902.329	0.030
	−0.004	16.1949	895.743	0.028
	−0.003	16.1946	895.739	0.028
	−0.003	16.1963	895.743	0.028
	−0.003	16.1913	895.741	0.028
	−0.005	7.1952	890.086	0.027
	−0.002	7.1967	890.085	0.027
	−0.003	7.1956	890.086	0.027
	−0.004	7.1966	890.086	0.027
	19.997	30.5609	887.684	0.031
	19.998	30.5565	887.681	0.031
	19.998	30.5587	887.682	0.031
	19.998	30.5568	887.681	0.031
	19.998	27.3354	885.675	0.031
	19.998	27.3308	885.671	0.031
	19.998	27.3307	885.672	0.031
	19.999	27.3330	885.674	0.031
	19.997	20.3391	881.181	0.029
	19.999	20.3376	881.176	0.029
	19.998	20.3372	881.180	0.029
	19.998	20.3362	881.179	0.029
	19.997	11.9792	875.520	0.028
	19.999	11.9806	875.519	0.028
	19.998	11.9781	875.520	0.028
	19.998	11.9771	875.519	0.028
	19.997	6.1452	871.356	0.027
	19.998	6.1447	871.353	0.027
	19.998	6.1441	871.355	0.027
	19.998	6.1451	871.356	0.027
	19.997	2.0015	868.280	0.027
	19.998	2.0056	868.281	0.027
	19.998	2.0063	868.281	0.027
	19.998	2.0078	868.281	0.027
	20.000	1.9983	868.276	0.027
	20.000	1.9973	868.274	0.027
	19.999	1.9996	868.277	0.027
	20.000	1.9994	868.274	0.027
	19.999	1.9992	868.274	0.027
	20.000	2.0000	868.275	0.027
	19.999	1.9993	868.276	0.027
	19.999	2.0022	868.278	0.027
	20.000	2.0004	868.277	0.027
	20.000	2.0025	868.277	0.027
	19.999	2.0039	868.280	0.027
	20.000	2.0029	868.278	0.027
	49.996	32.9841	864.927	0.035
	49.997	32.9820	864.927	0.035
	49.998	32.9772	864.920	0.035
	49.999	32.9801	864.922	0.035
	49.999	27.8278	861.270	0.034
	50.001	27.8253	861.266	0.034
	50.000	27.8228	861.265	0.034
	50.000	27.8218	861.265	0.034
	50.000	19.9628	855.405	0.032
	50.000	19.9585	855.403	0.032
	50.000	19.9575	855.399	0.032
	50.001	19.9590	855.400	0.032
	50.000	11.0857	848.311	0.031
	49.999	11.0829	848.309	0.031
	50.000	11.0831	848.307	0.031
	50.000	11.0782	848.304	0.031
	49.998	5.8874	843.881	0.030
	50.000	5.8869	843.880	0.030
	50.000	5.8849	843.882	0.030
	49.999	5.8838	843.879	0.030
	49.998	1.5804	840.035	0.030
	50.000	1.5816	840.036	0.030
	50.000	1.5807	840.035	0.030
	49.999	1.5819	840.036	0.030
	79.995	28.2844	837.014	0.038
	79.996	28.2827	837.012	0.038
	79.996	28.2826	837.010	0.038
	79.997	28.2848	837.011	0.038
	79.996	19.4690	829.354	0.036
	79.998	19.4694	829.352	0.036
	79.999	19.4665	829.347	0.036
	79.998	19.4640	829.347	0.036
	79.998	11.3224	821.647	0.035
	79.997	11.3192	821.639	0.035
	79.998	11.3200	821.643	0.035
	79.998	11.3199	821.640	0.035
	79.997	5.9936	816.209	0.034
	79.999	5.9937	816.206	0.034
	79.998	5.9954	816.207	0.034
	79.997	5.9945	816.208	0.034
	79.997	2.1535	812.055	0.034
	79.998	2.1546	812.057	0.034
	79.998	2.1556	812.055	0.034
	79.998	2.1525	812.052	0.034
	99.992	17.6003	810.191	0.038
	99.995	17.6029	810.195	0.038
	99.996	17.5995	810.189	0.038
	99.996	17.5996	810.188	0.038
	119.996	32.5440	808.391	0.043
	119.996	32.5433	808.393	0.043
	119.996	32.5427	808.389	0.043
	119.996	32.5412	808.385	0.043
	119.996	28.1025	803.966	0.042
	119.995	28.0980	803.961	0.042
	119.996	28.0996	803.959	0.042
	119.997	28.1017	803.961	0.042
	119.995	19.1983	794.384	0.040
	119.997	19.1966	794.381	0.040
	119.997	19.1978	794.382	0.040
	119.997	19.1966	794.381	0.040
	119.997	11.5355	785.196	0.039
	119.997	11.5347	785.196	0.039
	119.997	11.5340	785.194	0.039
	119.997	11.5360	785.198	0.039
	119.996	5.8395	777.639	0.039
	119.997	5.8405	777.643	0.039
	119.997	5.8384	777.636	0.039
	119.997	5.8373	777.637	0.039
	119.995	1.9513	772.036	0.038
	119.997	1.9553	772.039	0.038
	119.997	1.9557	772.039	0.038
	119.997	1.9562	772.040	0.038
	149.995	20.6286	769.625	0.043
	149.996	20.6304	769.627	0.043
	149.996	20.6320	769.629	0.043
	149.998	20.6329	769.629	0.043
	149.998	12.0061	757.332	0.042
	149.997	12.0055	757.335	0.042
	149.997	12.0043	757.331	0.042
	149.998	12.0047	757.331	0.042
Filling 1a				
	149.998	6.1527	747.768	0.041
	149.998	6.1525	747.773	0.041
	149.997	6.1517	747.772	0.041
	149.998	6.1511	747.767	0.041
	149.998	1.4862	739.185	0.041
	149.998	1.4859	739.185	0.041
	149.998	1.4858	739.186	0.041
	149.998	1.4858	739.184	0.041
	50.002	2.0528	840.504	0.030
	50.001	2.0540	840.506	0.030
	50.000	2.0528	840.506	0.030
	50.000	2.0523	840.507	0.030
	50.000	2.0493	840.503	0.030
	50.001	2.0499	840.502	0.030
	50.000	2.0474	840.501	0.030
	50.000	2.0467	840.499	0.030
	50.000	2.0469	840.500	0.030
	50.000	2.0441	840.498	0.030
	50.000	2.0472	840.500	0.030
	50.000	2.0436	840.497	0.030
